# Lower in vivo locus coeruleus integrity is associated with lower cortical thickness in older individuals with elevated Alzheimer’s pathology: a cohort study

**DOI:** 10.1186/s13195-024-01500-0

**Published:** 2024-06-17

**Authors:** Nina Engels-Domínguez, Elouise A. Koops, Stephanie Hsieh, Emma E. Wiklund, Aaron P. Schultz, Joost M. Riphagen, Prokopis C. Prokopiou, Bernard J. Hanseeuw, Dorene M. Rentz, Reisa A. Sperling, Keith A. Johnson, Heidi I. L. Jacobs

**Affiliations:** 1 The Athinoula A. Martinos Center for Biomedical Imaging, Department of Radiology, Massachusetts General Hospital, Harvard Medical School, Boston, MA 02129 USA; 2 Faculty of Health, Medicine and Life Sciences, School for Mental Health and Neuroscience, Alzheimer Centre Limburg, Maastricht University, Maastricht, The Netherlands; 3 Department of Neurology, Massachusetts General Hospital, Harvard Medical School, Boston, MA USA; 4 Gordon Center for Medical Imaging, Department of Radiology, Massachusetts General Hospital, Harvard Medical School, Boston, MA USA; 5 Department of Neurology, Cliniques Universitaires Saint-Luc, Brussels, Belgium; 6 Center for Alzheimer Research and Treatment, Department of Neurology, Brigham and Women’s Hospital, Harvard Medical School, Boston, MA USA

**Keywords:** Locus coeruleus, Cortical thickness, Neurodegeneration, Brain structure, MRI, PiB-PET, FTP-PET, Alzheimer’s disease

## Abstract

**Background:**

Autopsy work indicates that the widely-projecting noradrenergic pontine locus coeruleus (LC) is among the earliest regions to accumulate hyperphosphorylated tau, a neuropathological Alzheimer’s disease (AD) hallmark. This early tau deposition is accompanied by a reduced density of LC projections and a reduction of norepinephrine’s neuroprotective effects, potentially compromising the neuronal integrity of LC’s cortical targets. Previous studies suggest that lower magnetic resonance imaging (MRI)-derived LC integrity may signal cortical tissue degeneration in cognitively healthy, older individuals. However, whether these observations are driven by underlying AD pathology remains unknown. To that end, we examined potential effect modifications by cortical beta-amyloid and tau pathology on the association between in vivo LC integrity, as quantified by LC MRI signal intensity, and cortical neurodegeneration, as indexed by cortical thickness.

**Methods:**

A total of 165 older individuals (74.24 ± 9.72 years, ~ 60% female, 10% cognitively impaired) underwent whole-brain and dedicated LC 3T-MRI, Pittsburgh Compound-B (PiB, beta-amyloid) and Flortaucipir (FTP, tau) positron emission tomography. Linear regression analyses with bootstrapped standard errors (*n* = 2000) assessed associations between bilateral cortical thickness and i) LC MRI signal intensity and, ii) LC MRI signal intensity interacted with cortical FTP or PiB (i.e., EC FTP, IT FTP, neocortical PiB) in the entire sample and a low beta-amyloid subsample.

**Results:**

Across the entire sample, we found a direct effect, where lower LC MRI signal intensity was associated with lower mediolateral temporal cortical thickness. Evaluation of potential effect modifications by FTP or PiB revealed that lower LC MRI signal intensity was related to lower cortical thickness, particularly in individuals with elevated (EC, IT) FTP or (neocortical) PiB. The latter result was present starting from subthreshold PiB values. In low PiB individuals, lower LC MRI signal intensity was related to lower EC cortical thickness in the context of elevated EC FTP.

**Conclusions:**

Our findings suggest that LC-related cortical neurodegeneration patterns in older individuals correspond to regions representing early Braak stages and may reflect a combination of LC projection density loss and emergence of cortical AD pathology. This provides a novel understanding that LC-related cortical neurodegeneration may signal downstream consequences of AD-related pathology, rather than being exclusively a result of aging.

**Supplementary Information:**

The online version contains supplementary material available at 10.1186/s13195-024-01500-0.

## Background

Late-onset Alzheimer’s disease (AD), the most prevalent form of dementia, has historically been characterized by the stereotypical spatiotemporal progression of neuropathological beta-amyloid plaques and neurofibrillary tau tangles (NFTs) throughout the brain, often preceding the emergence of symptoms by decades [1, 2]. Autopsy work suggests that one of the first regions to harbor phosphorylated tau pathology is the pontine locus coeruleus (LC), the principal nucleus releasing norepinephrine (NE) to subcortical and widespread cortical targets [3, 4]. As outlined in the Braak staging scheme, accumulation of tau in the subcortical brainstem (Braak stage a-c) is followed by the entorhinal cortex (EC; Braak stage I-II), then the hippocampus (Braak stage III), after which it progresses to lateral temporal areas (Braak stage IV), and ultimately appears throughout the entire neocortex (Braak stage V-VI) [3]. Once NFTs are present in allocortical regions, beta-amyloid plaques can be robustly detected with positron emission tomography (PET) imaging [5, 6]. Together, these pathologies contribute to neurodegeneration or cortical atrophy [1].

Even though the LC accumulates tau early in adulthood, LC neurons are rather resilient and do not die until after Braak stage III-IV [7]. However, both neuropathology and animal studies reported that this early tau accumulation is associated with dendritic atrophy and loss of projections to the cortex, especially affecting projections to subcortical, hippocampal, and other neocortical regions [8, 9]. Despite that LC neurons are sturdy at this early stage, their functionality is impacted, possibly explaining the progression of tau to the medial temporal lobe. It is therefore likely that lower LC integrity will be associated with atrophy in the medial temporal lobe early on in the disease. With the emergence of cortical beta-amyloid and tau, and tau being more closely associated to neurodegeneration than beta-amyloid [10], it can be expected that these patterns will become more widespread and reflect the topography of Braak stages. Thus, the gradual loss of NE projections to the cortex and changes in the associated neuroprotective properties of NE, such as the modulation of neuroinflammation and clearance of toxins and beta-amyloid [11, 12], can have a negative impact on the neuronal integrity of the target regions of the LC. Examining the neurodegenerative changes associated with changes in LC integrity in older individuals along the continuum of AD can increase our understanding of the pathophysiologic consequences of early tau accumulation in the LC and provide insights into the role of the LC in the pathophysiologic cascade model of AD.

Previous work by Bachman, Dahl [13] revealed a positive association between magnetic resonance imaging (MRI)-derived LC integrity and cortical thickness in cognitively healthy, older individuals, as compared to younger individuals. Of note, the effect in older individuals remained in place when regressing out age effects. To the best of our knowledge, only one other study investigated similar associations in older male adults (age range: 62–71 years). Elman, Puckett [14] found no relation between LC integrity and cortical thickness that survived *FDR*-correction but did find that higher LC integrity was related to higher total hindered and lower free water diffusion, which reflects better microstructural health. Both Bachman, Dahl [13] and Elman, Puckett [14] provide evidence that changes in LC integrity may have consequences for cortical tissue and may be critical for healthy cognitive aging. However, it remains to be established whether these findings extend beyond healthy aging, as the contribution of underlying beta-amyloid and tau pathology have not yet been addressed. To that end, we associated LC integrity, as quantified by LC MRI signal intensity extracted from dedicated LC turbo-spin-echo scans, with cortical neurodegeneration, as indexed by cortical thickness, and investigated the potential effect modifications by beta-amyloid and tau pathology. Utilizing a subset of individuals with low beta-amyloid deposition allowed us to determine the association between LC integrity and the topography of neurodegeneration in the context of early tau deposition. Ultimately, these findings will be critical to understanding the impact of LC structure on AD-related neurodegeneration as well as its impact on effectively communicating with important higher-order cortical brain regions.

## Methods

### Participants

A total of 165 participants from the well-characterized, observational Harvard Aging Brain Study (HABS [15]) were included in this study. The ongoing, longitudinal HABS cohort aims to expand the field’s understanding of healthy aging and preclinical AD processes, and recruited to that end healthy individuals of age 50 and older with scores of 0 on the Clinical Dementia Rating (CDR [16]), below 11 on the Geriatric Depression Scale (GDS [17]), above 25 on the Mini-Mental State Examination (MMSE [18]), and within the education-adjusted norms of the Logical Memory Delayed Recall [19] at baseline. Exclusion criteria consisted of prevailing alcohol or substance abuse, head trauma, and report of physical or psychiatric illnesses. For the present study, only cross-sectional data from HABS participants who underwent 3T MRI, including a dedicated LC sequence, Pittsburgh Compound-B (PiB)-PET, and Flortaucipir (FTP)-PET scanning were selected. LC imaging was included mid-study, at which point some participants started showing cognitive decline. As such, approximately 10% (*n* = 16) of the utilized sample was classified as cognitively impaired based on CDR status. CDR status was assessed and reviewed for consensus by experienced clinicians. All participants provided informed consent for participation and received monetary compensation according to the regulations of the Mass General Brigham Institutional Review Board. Ethics approval for this study was obtained from the Mass General Brigham Institutional Review Board in accordance with the Belmont Report [20].

### Structural magnetic resonance imaging

Structural MRI data were collected at the Athinoula A. Martinos Center for Biomedical Imaging of Massachusetts General Hospital utilizing a Siemens 3.0T TIM-Trio scanner with a 12-channel phased-array head coil. A three-dimensional T1-weighted Magnetization-Prepared Rapid Acquisition Gradient-Echo (MPRAGE) structural image was obtained with the following parameters: Repetition Time (TR) = 2300; Echo Time (TE) = 2.95 ms; Inversion Time = 900 ms; Acquisition Time (TA) = 5:12 min; Flip Angle (FA) = 9°; Resolution = 1.1 × 1.1 × 1.2 mm; Acquisition Matrix = 270 × 254 × 212 mm; 176 slices per slab with sagittal orientation; double-factor GRAPPA acceleration. Our dedicated LC sequence is cost-effective, non-invasive, and has a fast acquisition and processing pipeline, making it a clinically easy-to-implement methodology. It consisted of a two-dimensional T1-weighted Turbo-Spin Echo (TSE) sequence that included magnetization transfer (MT) contrast (TR = 743 ms; TE = 16 ms; TA = 3:24 min; FA = 180°; Resolution = 0.4 × 0.4 × 3.00 mm; Acquisition Matrix = 208 × 166 × 22 mm; 6 slices with transversal orientation; 4 averages). Given the importance of minimal motion for proper visualization of the LC, the TSE sequence was acquired as close as possible in time to the MPRAGE, and participants were recurrently instructed to lay still. Head motion was restrained with foam pillows and extendable bumpers during all sequences, and participants were provided with ear plugs and headphones to protect hearing.

The structural MPRAGE image was processed with the standard automated reconstruction protocol of FreeSurfer (FS, version 6.0.0), which incorporates motion correction, Talairach registration, intensity normalization, skull-stripping, segmentation and parcellation (left versus (vs.) right hemispheres, cortical vs. subcortical regions, white vs. gray matter), as well as validation of topology and surface geometry [21]. Furthermore, all data were inspected manually with FS’s tkmedit-tool to ensure data quality and corrected if necessary.

LC MRI signal intensity was calculated as described in Jacobs, Becker [22]. In brief, four equally-sized boxes that functioned as search limits were placed bilaterally on the locus coeruleus (corroborated by overlaying with a validated LC-template [23]) and pontine tegmentum on the Montreal Neurological Institute (MNI) template, with the latter region serving as reference region. After warping the MNI template and registering each LC scan to the participant’s native T1, the four equally-sized boxes were warped to the T1 as well. Subsequently, signal intensities corresponding to the LC were normalized against the average intensity of the reference region. Then, LC signal intensity was established by searching for clusters of 5 connecting voxels with the highest average signal intensities in an iterative fashion (*n* = 30). Left and right LC signal intensity values were averaged to create a bilateral composite for analyses, as previous autopsy work has not found asymmetry in LC neuronal population or tau deposition [24–27].

**Fig. 1:**
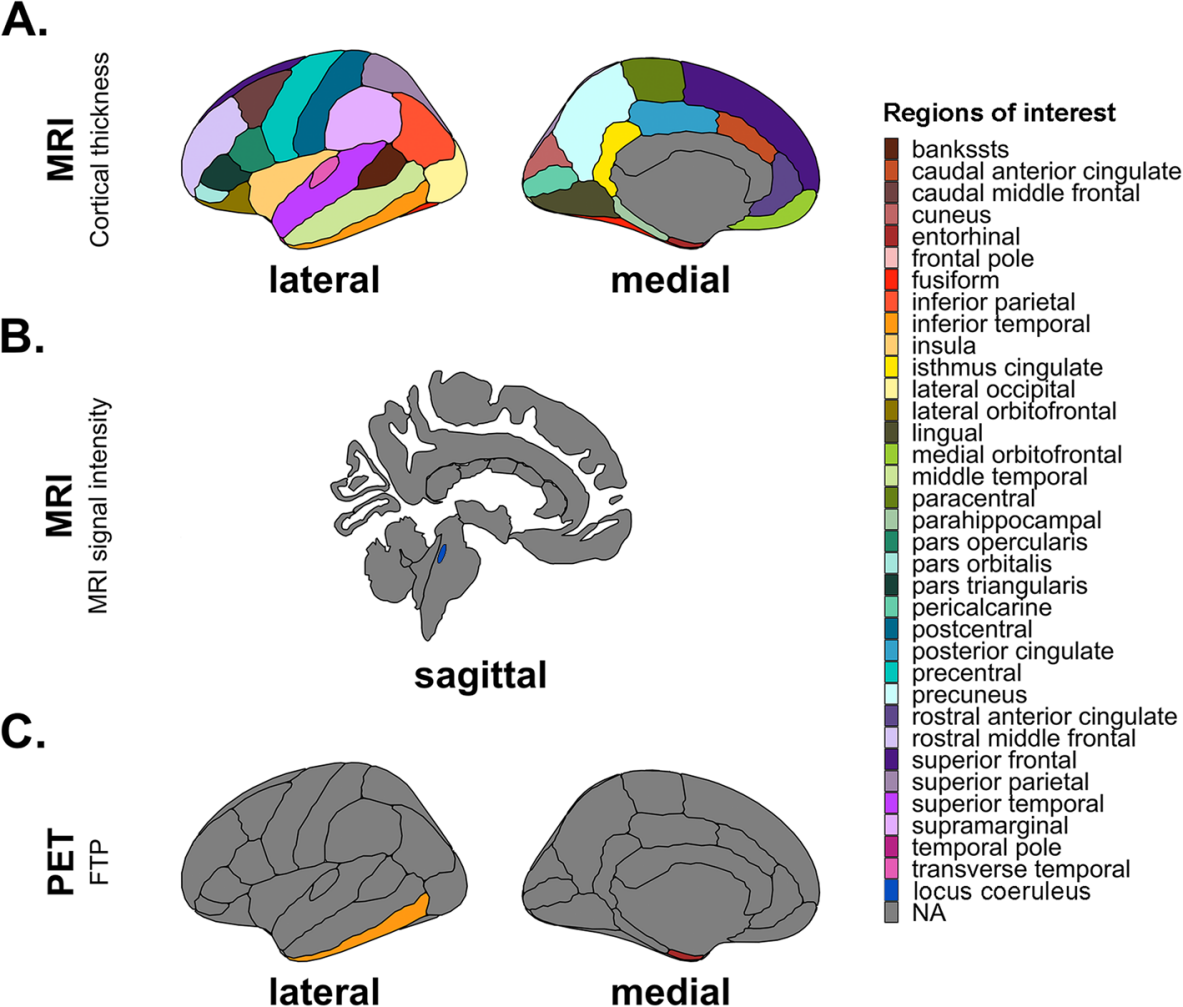
Regions of interest depicted per modality and measure of interest. Regions of interest for **A** MRI cortical thickness, **B** MRI signal intensity, **C** PET-FTP measures. Cortical ROIs are based on the Desikan-Killiany atlas. Abbreviations: Bankssts, banks of superior temporal sulcus; ROIs, regions of interest

Unilateral cortical thickness values corresponding to 34 different cortical regions labeled according to the Desikan-Killiany atlas were extracted with FS [28], after which these values were averaged across hemispheres to obtain regional bilateral values. Overall regions of interest (ROIs) were limited to the LC and the cortical thickness regions depicted in Fig. 1.

### Positron emission tomography imaging

PiB- and FTP-PET data were acquired at Massachusetts General Hospital utilizing a Siemens ECAT EXACT HR+ PET scanner as described previously [22, 29, 30]. For the PiB tracer, bolus injections of 8.5–15 mCi were administered, after which data was obtained dynamically in a window of 60 min (twelve 15-s frames, fifty-seven 1-min frames). For the FTP tracer, bolus injections of 9–11 mCi were administered, and data was acquired after 75–105 min (four 5-min frames). To reconstruct the data, standard data corrections were employed. Additionally, all data frames were inspected manually to ensure data quality.

Tracer retention was examined by co-registering the PET data to the participants’ MPRAGE image and transforming FS ROIs into the native PET space. Cerebellar gray served as reference tissue for both tracers, and all data were corrected for partial volume (PVC) effects with FS’s Geometric Transfer Matrix approach assuming a uniform 6 mm point spread function [31]. By means of the Logan graphical method, PiB-PET data was expressed with distribution volume ratio (DVR). Neocortical PiB consisted of a combination of frontal, lateral temporal, and retrosplenial cortices (FLR) values, and functioned as the basis for beta-amyloid status classification in reference to the PiB cutoff value of 1.324 DVR (PVC), which was determined a priori in the entire HABS cohort using Gaussian Mixture Modelling [32]. About 32% (*n* = 53) of the sample was considered to have elevated beta-amyloid based on the PiB cut-off value (for non-PVC PiB the cut-off was determined at 1.20 DVR resulting in *n* = 46 with elevated beta-amyloid), and 68% (*n* = 112) to have low beta-amyloid. FTP-PET data was expressed as the standardized uptake value ratio (SUVr). Consistent with previous work focusing on preclinical AD [33–35], regions of interest were limited to EC and inferior temporal (IT) FTP representing earlier and later stages of cortical tau burden (Fig. 1). PiB- and FTP-PET sessions were generally completed on the same day (median: 0 days, interquartile range (IQR): 0 to 0 days). Median time differences between MRI imaging and PET imaging sessions were 12 days for PiB-PET (IQR: -21 to 55 days) and 20 days for FTP-PET (IQR: -18 to 62 days).

### Statistical analyses

All statistical analyses were conducted using RStudio (version 1.2.5033, R version 3.6.3). Group differences were assessed using t-tests for normal continuous variables, Kruskal–Wallis tests for non-normal continuous variables, and chi-squared tests for categorical variables. Given the heteroskedastic nature of the data, associations were assessed utilizing linear regression analyses with bootstrapped standard errors (*n* = 2000). First, associations between bilateral cortical thickness and LC MRI signal intensity (5 voxel clusters) were examined in the entire sample. Then, to assess potential effect modifications by pathology, we evaluated the interaction between LC MRI signal intensity and a continuous measure of pathology (i.e., EC FTP, IT FTP, neocortical PiB) on bilateral cortical thickness values in the entire sample. Floodlight analyses were conducted to identify the lower bounds of the significance range of the effect modification by neocortical PiB. All models included age, sex, and education as covariates and were corrected for multiple comparisons using *FDR-*correction. Regions of cortical thickness showing significant relationships across models were categorized into aggregates representative of early and later Braak staging for visualization purposes: the EC represents cortical thickness (CT) in the Braak I-II region, and an aggregate of the IT, middle temporal, and fusiform cortex represents CT in Braak IV regions [36]. Lastly, analyses were repeated in the low beta-amyloid subsample. Additional sensitivity analyses were performed using dichotomous neocortical PiB status (instead of continuous neocortical PiB), non-PVC data, and the CDR = 0 subsample. PVC and non-PVC analyses were compared, and congruency was assessed by calculating the percentage of significant regions detected in the non-PVC analysis that overlapped with the significant regions identified in the PVC analysis. Post-hoc power calculations based on effect sizes reported in Bachman, Dahl [13] for the main effect models and estimated effect sizes for the interactive effect models were conducted to verify sample sizes [37]. For the main effect models, a minimum sample size of *n* = 80 was required to detect a Cohen’s f^2^ effect of at least 0.16 (medium effect size; based on R^2^ of the most similar analysis reported in Bachman, Dahl [13]) with a two-tailed alpha of 0.05 and a power of 0.80. For the interactive effect models, a sample size of minimum *n* = 98 was required to detect a Cohen’s f^2^ effect of at least 0.15 (medium effect size; based on estimations as to date, no other reports are available) with a two-tailed alpha of 0.05 and a power of 0.80.

## Results

### Samples characteristics

The sample of HABS participants (*n* = 165) had an average age of 74.24 years (median: 75, standard deviation: 9.72, range: 50 to 94 years), an average education of 16.35 years (median: 16, range: 9 to 20 years) and consisted of 98 females (~ 60%; Table 1). The subsample of low beta-amyloid participants (*n* = 112) was similar to the entire sample with respect to the proportion of sex (females: 55%) and educational level (mean: 16.31 years, median: 16, range: 9 to 20 years), but was significantly younger (mean: 71.85 years, median: 72.88, standard deviation: 9.67, range: 50 to 91 years), had less APOE-ε4 carriers and fewer participants with a CDR > 0 score. As expected, the low beta-amyloid sample also displayed lower FTP- and PiB-PET values, as well as higher LC MRI signal intensity and MMSE values compared to participants with elevated beta-amyloid. No significant differences were observed in sex or race proportions. The CDR = 0 sample used for sensitivity analyses was similar to the main sample (Supplemental Table 1).

**Table 1:** Characteristics of participants sorted on PiB status

Characteristics of participants sorted on PiB status			
	*Low beta-amyloid*	*Elevated beta-amyloid*	*P-value*
n	112	53	
Age	71.85 (9.67)	79.30 (7.74)	< 0.001 **
Sex, No. (%) = M	45 (40.2)	22 (41.5)	1.000
Years of Education	16.31 (2.87)	16.42 (2.92)	0.832
Race, No. (%)			0.994
AS	3 (2.7)	1 (1.9)	
B	12 (10.7)	6 (11.5)	
NH	17 (15.2)	8 (15.4)	
Other	2 (1.8)	1 (1.9)	
Unknown	4 (3.6)	1 (1.9)	
W	74 (66.1)	35 (67.3)	
APOE-ε4 Carrier Status, No. (%) = ε4+	13 (12.0)	27 (52.9)	< 0.001 **
LC MRI signal intensity	1.34 (0.05)	1.30 (0.03)	< 0.001 **
IT FTP values (SUVr, PVC)	1.41 (0.16)	1.66 (0.42)	< 0.001 **
EC FTP values (SUVr, PVC)	1.28 (0.26)	1.56 (0.43)	< 0.001 **
FLR-PiB values (DVR, PVC)	1.19 (0.06)	1.93 (0.47)	< 0.001 **
Clinical Dementia Rating, No. (%)			0.017 *
0	106 (94.6)	43 (81.1)	
0.5	6 (5.4)	9 (17.0)	
1	0 (0.0)	1 (1.9)	
MMSE Score	29.04 (1.26)	28.08 (1.99)	< 0.001 **

Data is presented as numbers and (percentages) or means and (standard deviations). Chi-square tests and Kruskal tests were conducted to reveal group differences for categorical and non-normal continuous variables, respectively. Missing data for Race (*n* = 1) and APOE-ε4 Carrier Status (*n* = 6). Abbreviations: No, number; M, male; AS, Asian; B, Black or African American; NH, Native Hawaiian or other Pacific Islander; W, White; LC, locus coeruleus; IT, inferior temporal; FTP, [^18^F]-flortaucipir; SUVr, standardized uptake value ratio; PVC, partial-volume corrected; EC, entorhinal cortex; FLR, frontal, laterotemporal and retrosplenial cortices; PiB, [^11^C]-Pittsburgh Compound-B; DVR, distribution volume ratio; MMSE, Mini-Mental State Examination. *P < .05; **P < .001

### Lower LC MRI signal intensity is associated with lower mediolateral temporal cortical thickness

**Fig. 2:**
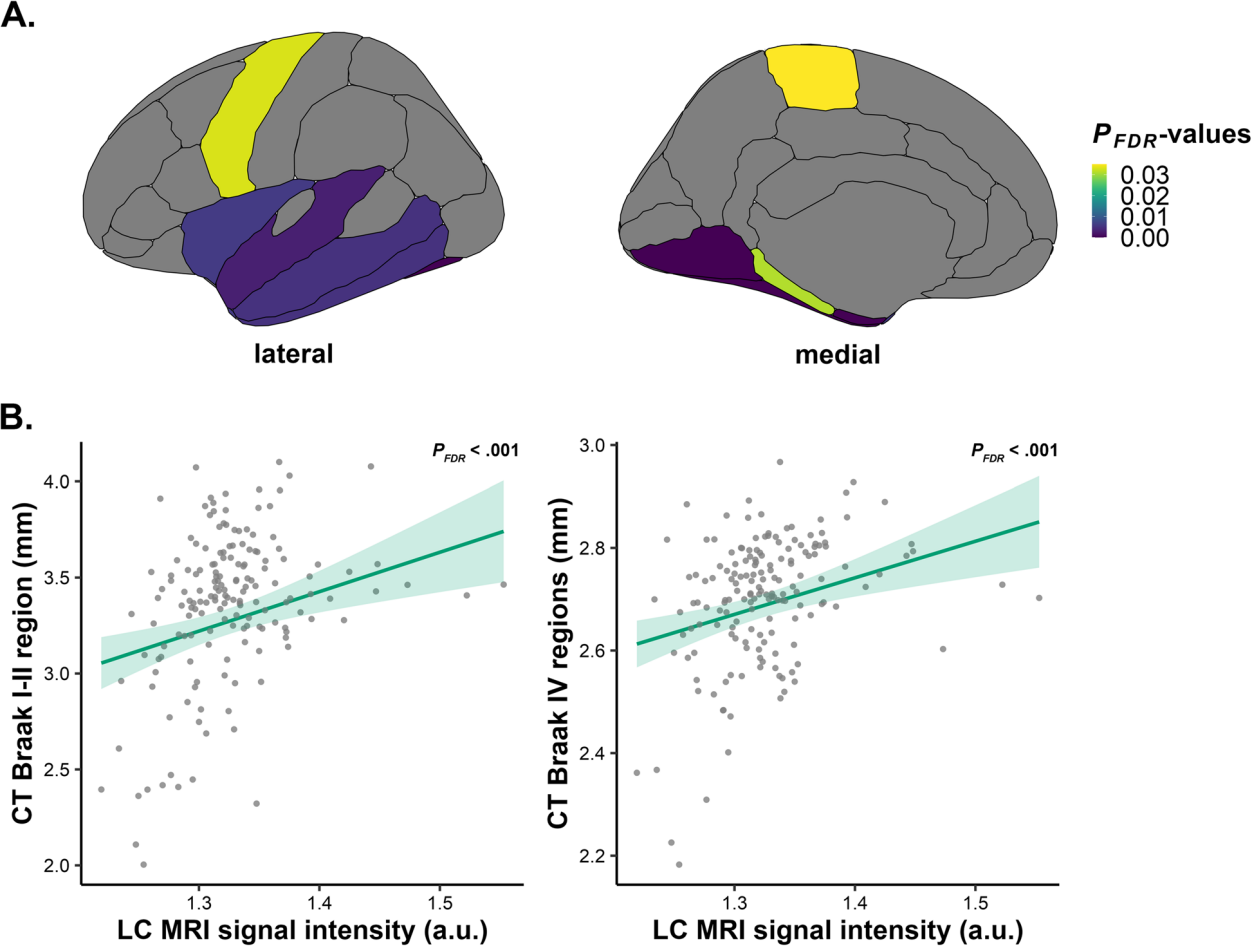
Lower LC MRI signal intensity is associated with lower mediolateral temporal cortical thickness. Associations between measures of cortical thickness and LC MRI signal intensity for the entire sample, corrected for age, sex, and years of education. **A** Positive associations and corresponding test statistics between CT regions (entorhinal, fusiform, inferior temporal, lingual, middle temporal, parahippocampal, paracentral, precentral, superior temporal, temporal polar and insular cortices) and LC MRI signal intensity. **B** Visualization of the association between CT of the Braak I-II region (left; *n* = 165; *P*_*FDR*_ < .001) as well as CT of Braak IV regions (right; *n* = 165; *P*_*FDR*_ < .001) and LC MRI signal intensity: lower LC MRI signal intensity is associated with lower CT in both early Braak I-II region and late Braak IV regions. Shaded areas represent the 95% confidence interval. Abbreviations: LC, locus coeruleus; CT, cortical thickness

Across the entire sample, we observed that lower LC MRI signal intensity was associated with lower cortical thickness in the entorhinal, fusiform, inferior temporal, lingual, middle temporal, parahippocampal, paracentral, precentral, superior temporal, temporal polar and insular cortices, (*n* = 165; CT Braak I-II region: *R*^*2*^ = 0.27, *B* = 2.055, 95% CI [0.879, 3.253], *P*_*FDR*_ < .001; CT Braak IV regions: *R*^*2*^ = 0.19, *B* = 0.713, 95% CI [0.324, 1.090], *P*_*FDR*_ < .001; Fig. 2; please refer to Supplemental Table 2 for statistics of individual regions). Similar results were observed when correcting for neocortical PiB, either continuous or dichotomous (*n* = 165; Supplemental Tables 3–4). No significant associations between LC MRI signal intensity and cortical thickness were observed in the individuals with low beta-amyloid (*n* = 112; Supplemental Table 5). Sensitivity analyses in the entire dataset using non-PVC PiB-data (continuous or dichotomous PiB) as a covariate revealed an average congruency of ~ 100% in terms of anatomy and number of regions involved (Supplemental Tables 3–4). Within the low beta-amyloid sample, the non-PVC results were largely congruent to the PVC results with additional significant associations for the EC and lingual cortices. It should be noted that these regions showed marginal significance in the PVC analyses (Supplemental Table 5). Analyses on the CDR = 0 subsample revealed comparable positive associations for the key regions that were detected in the entire sample (*n* = 149; Supplemental Table 6).

### Lower LC MRI signal intensity is related to lower cortical thickness, particularly in individuals with elevated pathology

We then aimed to assess whether pathology modifies the association between LC MRI signal intensity and cortical thickness. First, we interacted LC MRI signal intensity with EC FTP: we found that lower LC MRI signal intensity is associated with lower cortical thickness of the entorhinal, fusiform, inferior parietal, inferior temporal, lateral occipital, lingual, middle temporal, parahippocampal, precentral and insular cortices, and that this association is stronger in individuals with elevated EC FTP (*n* = 165; CT Braak I-II region: *R*^*2*^ = 0.48, *B* = 4.233, 95% CI [2.345, 6.242], *P*_*FDR*_ < .001; CT Braak IV regions: *R*^*2*^ = 0.31, *B* = 1.284, 95% CI [0.599, 2.030], *P*_*FDR*_ < .001; Fig. 2A-B; please refer to Supplemental Table 7 for statistics of individual regions). Interacting LC MRI signal intensity with IT FTP instead or including continuous PiB as a covariate in both FTP models showed similar results (Supplemental Tables 8–10). Sensitivity analyses using non-PVC data revealed an average anatomical overlap of ~ 95% across these models (Supplemental Tables 7–10). Analyses on the CDR = 0 subsample showed positive associations between LC MRI signal intensity and entorhinal and fusiform cortical thickness, particularly in individuals with elevated EC FTP. These regions overlap with our findings in the entire sample, and in addition, we found positive associations for the isthmus cingulate and temporal polar cortical thickness (*n* = 149; Supplemental Table 11).

**Fig. 3:**
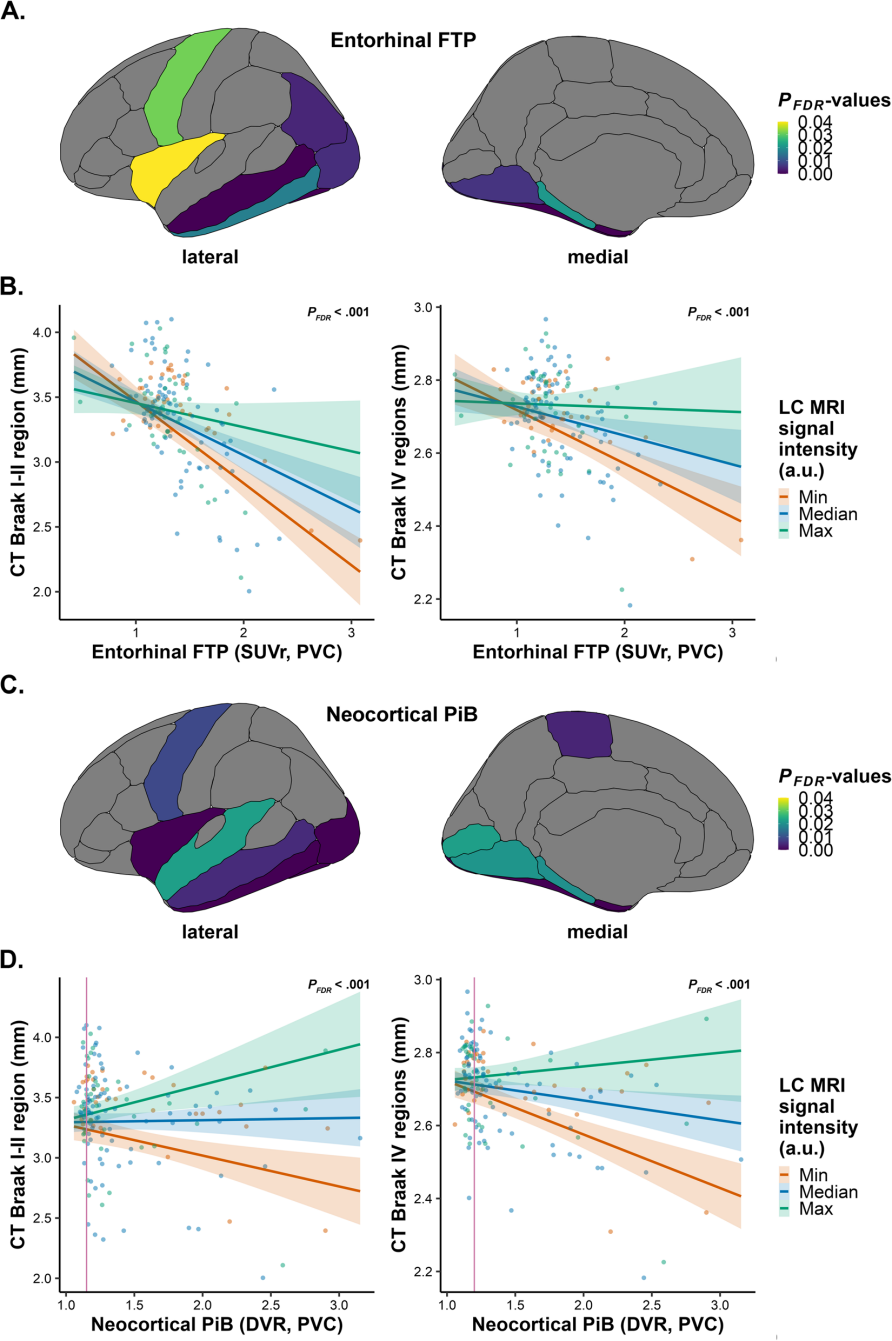
Lower LC MRI signal intensity is associated with lower cortical thickness, particularly in individuals with elevated pathology. Associations between measures of cortical thickness and LC MRI signal intensity interacted with continuous EC FTP or neocortical PiB for the entire sample, corrected for age, sex, and years of education. **A, C** Positive associations and corresponding test statistics between CT regions and the interaction of EC FTP (**A**; entorhinal, fusiform, inferior parietal, inferior temporal, lateral occipital, lingual, middle temporal, parahippocampal, precentral and insular cortices) or neocortical PiB (**C**; entorhinal, fusiform, inferior temporal, lateral occipital, lingual, middle temporal, parahippocampal, paracentral, pericalcarine, precentral, superior temporal and insular cortices) with LC MRI signal intensity. **B, D** Visualization of the association between CT of the Braak I-II region (left; *n* = 165; *P*_*FDR*_ < .001) as well as CT of Braak IV regions (right; *n* = 165; *P*_*FDR*_ < .001) and EC FTP (**B**) or neocortical PiB (**D**) interacted with LC MRI signal intensity: lower LC MRI signal intensity is associated with lower CT in both early Braak I-II region and late Braak IV regions, particularly in individuals with elevated EC FTP or neocortical PiB. As represented by the pink vertical lines in **D,** floodlight analyses reveal significance starting from subthreshold PiB values (left: ≥ 1.15; right: ≥ 1.20), as compared to the HABS cut-off: 1.324 DVR PVC. LC MRI signal intensity is shown at simple slopes representing minimum, median, and maximum for visualization, but analyses have been conducted continuously. Shaded areas represent the 95% confidence interval. Abbreviations: LC, locus coeruleus; CT, cortical thickness

Then, we assessed the potential effect modification of continuous neocortical PiB and observed that in individuals with elevated neocortical PiB, lower LC MRI signal intensity was associated with lower cortical thickness of the entorhinal, fusiform, inferior temporal, lateral occipital, lingual, middle temporal, parahippocampal, paracentral, pericalcarine, precentral, superior temporal and insular cortices (*n* = 165; CT Braak I-II region: *R*^*2*^ = 0.32, *B* = 5.199, 95% CI [2.514, 8.078], *P*_*FDR*_ < .001; CT Braak IV regions: *R*^*2*^ = 0.32, *B* = 1.749, 95% CI [0.896, 2.630], *P*_*FDR*_ < .001; Fig. 3C-D; Supplemental Table 12). To identify the lower bound of the significance range of the effect modification by PiB, flood light analyses demonstrated that associations between LC MRI signal intensity and cortical thickness start at subthreshold PiB values (CT Braak I-II region: ≥ 1.15; CT Braak IV regions: ≥ 1.20; Fig. 3D; HABS cut-off: 1.324 DVR PVC). Covarying for EC FTP demonstrated identical results (Supplemental Table 13). Running the two-way interaction analyses with dichotomous PiB revealed comparable results but did not include occipital lobe regions (Supplemental Table 14). Sensitivity analyses utilizing non-PVC data showed an anatomic overlap of ~ 96% for continuous PiB and of ~ 27% for models with dichotomous PiB, although overlap for the latter model was above 64% when considering reaching marginal significance in the non-PVC-analyses (Supplemental Tables 12–14). It should also be noted that the categorization of individuals into PiB groups changes slightly when utilizing the non-PVC threshold for dichotomous PiB (elevated beta-amyloid: ~ 28% (*n* = 46) of the sample). For the CDR = 0 subsample, continuous neocortical PiB did not moderate the relationships between LC MRI signal intensity and cortical thickness (*n* = 149; Supplemental Table 15).

### In individuals with low beta-amyloid, lower LC MRI signal intensity is related to lower entorhinal cortical thickness in the context of elevated EC FTP

**Fig. 4:**
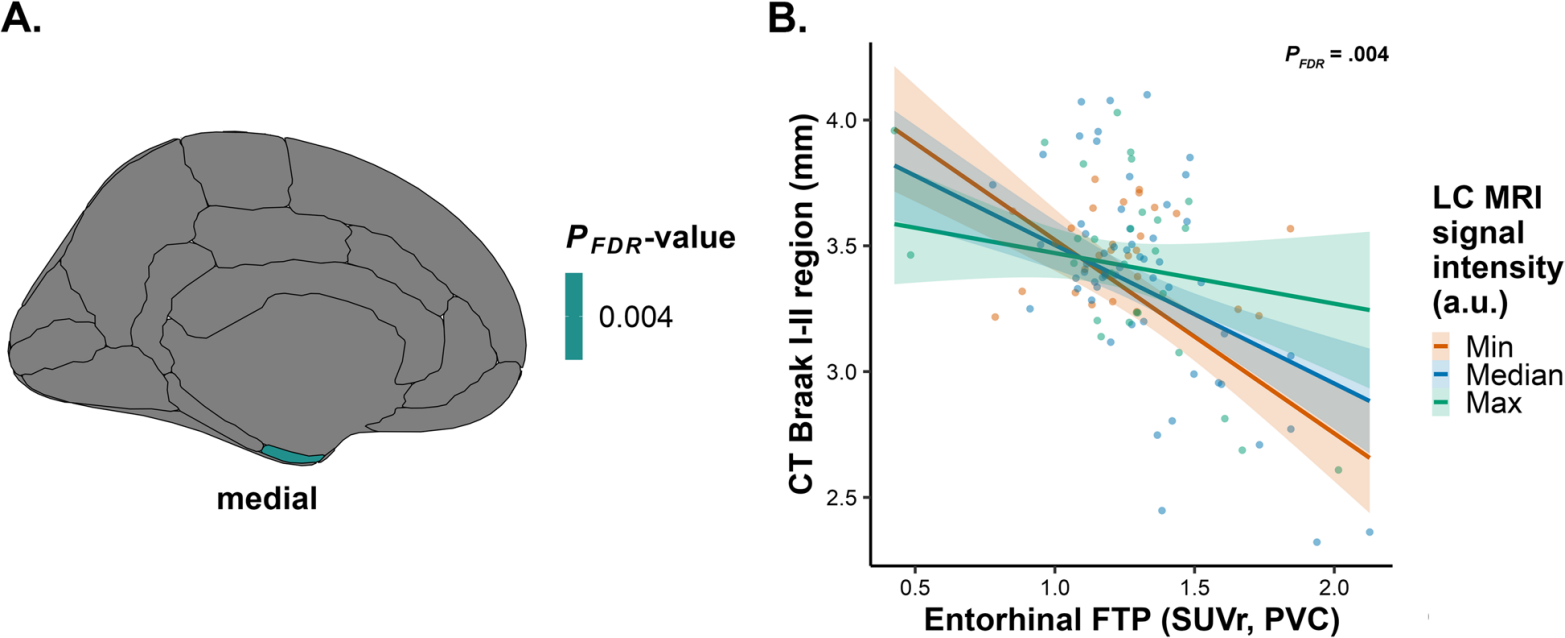
For the low beta-amyloid sample, lower LC MRI signal intensity is associated with lower entorhinal cortical thickness, particularly in individuals with elevated EC FTP.Associations between measures of cortical thickness and LC MRI signal intensity interacted with EC FTP for the low beta-amyloid sample, corrected for age, sex, and years of education.** A** Positive association and corresponding test statistic between CT region (entorhinal cortex) and the interaction of EC FTP with LC MRI signal intensity.** B** Visualization of the association between CT of the Braak I-II region (*n* = 112; *P*_*FDR*_ = .004) and EC FTP interacted with LC MRI signal intensity for the low beta-amyloid sample: lower LC MRI signal intensity is associated with lower CT in early Braak I-II region, particularly in individuals with elevated EC FTP. LC MRI signal intensity is shown at simple slopes representing minimum, median, and maximum for visualization, but analyses have been conducted continuously. Shaded areas represent the 95% confidence interval. Abbreviations: LC, locus coeruleus; CT, cortical thickness

Lastly, we examined the potential effect modification of FTP on the association between LC MRI signal intensity and cortical thickness in the subsample of low beta-amyloid individuals. We found that lower LC MRI signal intensity is related to lower entorhinal cortical thickness in individuals with low levels of neocortical PiB, in the context of elevated levels of EC FTP (*n* = 112; CT Braak I-II region: *R*^*2*^ = 0.41, *B* = 5.419, 95% CI [2.516, 8.160], *P*_*FDR*_ = .004; Fig. 4; Supplemental Table 16). Interacting LC MRI signal intensity with IT FTP showed the same results (Supplemental Table 17). Sensitivity analyses employing non-PVC data were similar (100% anatomical overlap; Supplemental Tables 16–17). Analyses on the CDR = 0 subsample with low beta-amyloid deposition similarly showed positive associations for entorhinal cortical thickness, and additional positive associations for the isthmus cingulate, lateral occipital, and temporal polar cortical thickness, particularly in individuals with elevated EC FTP (*n* = 112; Supplemental Table 18).

## Discussion

Previous studies demonstrated the importance of LC integrity within the context of healthy cognitive aging and its contributions to age-related changes in cortical tissue [13, 14]. To date, however, the question remains as to whether these findings extend beyond healthy cognitive aging and whether they may be moderated by underlying latent AD pathology, as AD pathology starts to accumulate two to three decades before clinical symptoms are evident. AD is characterized by the accumulation of beta-amyloid plaques and neurofibrillary tau tangles, which progressively spread through the brain and jointly promote cortical neurodegeneration. As autopsy studies reported that the LC is one of the initial regions to accumulate tauopathy and recent LC imaging studies reported that AD-related changes correlate with LC integrity [3, 38–40], gaining insights into the relationship between MRI-derived LC integrity and AD-related neurodegeneration will aid early detection and our understanding of AD’s disease progression. To that end, we examined the association between in vivo LC integrity and cortical neurodegeneration, and the potential effect modifications by cortical beta-amyloid and tau pathology.

Utilizing 3T MRI and PET data, we present robust and novel evidence from several (sensitivity) analyses that structural LC properties are associated with AD-related cortical neurodegeneration, starting from the early Braak stages. More specifically, our findings show that lower LC integrity is related to greater cortical atrophy in regions representative of both early Braak I-II and late Braak IV regions, particularly among individuals exhibiting elevated beta-amyloid and tau pathology. The association of LC integrity with cortical neurodegeneration started to emerge at subthreshold global beta-amyloid values in regions representative of Braak I-II regions. Importantly, when restricting the sample to individuals with low beta-amyloid pathology levels, the relationship between LC integrity and cortical (entorhinal) neurodegeneration was only observed in the presence of elevated entorhinal tau pathology. To our knowledge, few studies have reported on the relationship between LC integrity and cortical tissue [13, 14]. These studies reported that lower LC integrity was associated with cortical neurodegeneration in older individuals but did not take into account the potential presence of underlying AD pathology. Our findings add an important new perspective on previous studies in aging, as they suggest that LC-related neurodegeneration is not merely age-related but requires the contribution of initial cortical pathology. Overall, this contributes to the field’s current understanding of the pathogenesis of AD, and indicates that LC integrity may facilitate the identification of individuals at risk for AD.

Our findings are in line with previous post-mortem work demonstrating a linear decrease of approximately 8% in LC volume starting at Braak stage 0, which occurs as a function of pathology rather than age. This LC volume loss in the early Braak stages is assumed to represent a reduction of dendritic branching and of the projection density to the cortex [7, 8, 41]. In transgenic TgF344-AD rats expressing mutant amyloid precursor protein, presenilin-1, and age-dependent endogenous hyperphosphorylated tau in the LC, accumulation of tau in the LC resulted in decreased NE signaling and overall dysregulated NE as well as reductions in LC fiber density to the cortex [9]. Considering that the LC innervates the cortex extensively and releases NE, a neuromodulator with neuroprotective properties, compromised noradrenergic neurotransmission possibly resulting from tauopathy in the LC and associated changes in LC projection density could constitute part of the underlying mechanism of the association between diminished LC integrity and the degeneration of cortical structure. Both *in* and ex vivo human studies have shown that NE metabolism is upregulated in the initial disease stages, which may explain the manifestation of early neuropsychiatric symptoms; whereas NE metabolic levels appear to drop in late disease stages, most likely contributing to the behavioral and cognitive deficits observed later on in the continuum [41–54]. Furthermore, in transgenic mice dysregulated noradrenergic signaling to higher-order regions has also been associated with loss of its neuroprotective effects and reductions in beta-amyloid clearance [55–57], which together can promote cortical neuronal loss [58, 59]. A study by Chalermpalanupap, Schroeder [60] supports this notion by demonstrating that P301S tau transgenic mice with LC lesion-related NE depletion show hippocampal neurodegeneration.

Although we did not directly assess NE properties in this work, a study by van Hooren, Verhey [61] reported comparable findings. They demonstrated that higher noradrenergic turnover in concert with elevated beta-amyloid and tau pathology was related to cortical neurodegeneration in a sample of subjective cognitive decline, mild cognitive impairment (MCI), and AD patients. In contrast to our results, they did not observe a direct association between noradrenergic turnover and cortical thickness, possibly because our LC integrity measure reflects structural properties of the LC, which is likely closer in nature to neurodegeneration than the noradrenergic metabolite measure. Additionally, the sample of the van Hooren, Verhey [61] study spanned different stages of cognitive impairment, whereas our study focused predominantly on preclinical AD. Future studies should consider combining LC integrity with cerebrospinal fluid NE measures to disentangle the temporal dynamics of noradrenergic metabolism, LC structure, and cortical neurodegeneration along the AD continuum.

The spatial LC-related cortical thinning pattern observed in our results aligns well with the mediolateral temporal topography of initial Braak stages [36]. However, we also observed LC-related neurodegeneration in additional regions more representative of later Braak stages. One observed region is the insular cortex, typically classified as Braak stage IV [36]. The insula received relatively limited attention in the field of tau PET staging, despite its pivotal role as a central hub engaged in cognition, emotion processing, and decision-making, as well as its connection to the LC [62, 63]. Nonetheless, insular atrophy has been reported previously in patients with MCI as well as AD [63–66]. The insula has a high metabolic demand and widespread connections, including strong connections to the EC and limbic system, and may therefore be particularly vulnerable to pathology [63, 67]. Alternatively, the insular cortex is an integral component of the salience network, serving functions in which the LC is heavily implicated, and altered connectivity within this network has been detected previously in AD [68, 69].

We did not expect that LC integrity would be associated with cortical thickness in the precentral gyrus in individuals with elevated levels of AD pathology. Previous work reported presence of precentral gyrus atrophy in individuals with non-amnestic MCI (who tend to progress to other dementia types) or late AD [70]. However, work by Gordon, McCullough [71] assessing the relationship between tau pathology and neurodegeneration in a sample of 178 individuals (156 cognitively healthy, 22 mild dementia) showed cross-sectional tau-related cortical thinning of small clusters within the primary motor cortex. In both our analyses and the work by Gordon, McCullough [71], these precentral-related findings may reflect contributions from individuals on a faster disease trajectory. Prior research observed atrophy in the precentral cortex in asymptomatic older individuals who developed MCI or AD 5–10 years after imaging [72]. Further investigation is needed to identify factors contributing to the heterogeneity of atrophy patterns in different stages across the trajectory of AD.

The results of this study are bound by several limitations. Firstly, LC integrity was assessed through hyperintense LC MRI signal contrast assumed to reflect processes related to neuromelanin cell density [23]. However, the exact biological source of this signal remains subject of debate, as studies found other signal-contributing elements (i.e. lipids, water) [73–76]. Our findings indicate associations between lower LC integrity and cortical thinning, which exacerbate in the context of AD pathology; but it remains unclear whether LC contrast changes are more closely related to tau deposition or atrophy. Crucially, previous research by Jacobs, Becker [22] showed that both in vivo LC integrity and post-mortem LC tangle density – and not LC neuronal density — relate to initial cortical tau deposition, thereby cautiously alluding to a role for tau-related processes. Future work should investigate whether LC integrity predicts future rises in neocortical AD pathology and provide additional insights into the mechanisms that ultimately drive some individuals towards cognitive decline. Understanding these relationships could unveil who may be eligible for early prevention intervention trials aimed at delaying the onset of AD. Secondly, the MRI measures of both LC integrity and cortical atrophy were assessed at a single, concurrent time point, which limits inferences about the temporal nature of the association. Repeated measurements might shed light on the temporal dynamics of the positive associations found between LC integrity and cortical thickness. Similarly, we cannot preclude primary age-related tauopathy [77] for individuals with low beta-amyloid levels, since these individuals are still relatively young and might develop beta-amyloid and tau pathology in the future. Furthermore, to exclude age effects, we corrected all our analyses for age. Lastly, the sample consisted primarily of highly educated, white, and clinically healthy participants, which may reduce the generalizability of our findings to both broader and patient populations. Nonetheless, by focusing on a predominantly preclinical sample, we aimed to capture the earliest preclinical effects of the LC on neurodegeneration. Our sensitivity analyses on the CDR = 0 sample showed similar tau-related patterns, but not for PiB. Thus, these findings confirm that neurodegeneration is more closely associated with tau than beta-amyloid [10, 71], and that LC-related neurodegeneration can be detected in the earliest stages of the disease. Future studies should strive to obtain a more heterogeneous sample that includes a broader range of racial, ethnic, and educational backgrounds to allow for better overall generalizability.

## Conclusions

To conclude, we investigated the association between in vivo LC integrity and cortical thickness in older individuals within the context of AD pathology. Our findings show that lower LC integrity is associated with cortical neurodegeneration with a topography that is consistent with early Braak staging. As expected, these effects were more pronounced in individuals with elevated AD pathology, and importantly, in individuals with low levels of beta-amyloid, we only detected LC-related neurodegeneration in the presence of elevated entorhinal tau burden. This suggests that downstream cortical neurodegeneration is related to a combination of LC projection density loss and the emergence of cortical AD pathology. Altogether, these observations offer new insights into our current understanding of the LC in the pathophysiologic cascade of AD by highlighting for the first time that LC-related cortical neurodegeneration in older individuals may reflect the downstream consequences of AD-related pathology, rather than being solely a result of aging.

### Supplementary Information

Supplementary Material 1.

## Data Availability

The Harvard Aging Brain Study project is committed to publicly releasing its data. Baseline structural MRI, PiB-PET, FTP-PET, and cognitive follow-up data until year 5 are publicly available to the research community at https://habs.mgh.harvard.edu. Requests for material, currently available raw and processed data for all the datasets used in the study, and correspondence can be addressed to Dr. Sperling. Qualified investigators must abide by the Harvard Aging Brain Study online data use agreement, designed to protect the privacy of our participants.

## Abbreviations

AD: Alzheimer’s disease
NFT: Neurofibrillary tau tangle
LC: Locus coeruleus
NE: Norepinephrine
EC: Entorhinal cortex
PET: Positron emission tomography
MRI: Magnetic resonance imaging
HABS: Harvard Aging Brain Study
CDR: Clinical Dementia Rating
GDS: Geriatric Depression Scale
MMSE: Mini-Mental State Examination
PiB: Pittsburgh Compound-B
FTP: Flortaucipir
MPRAGE: Magnetization-Prepared Rapid Acquisition Gradient-Echo
TR: Repetition Time
TE: Echo Time
TA: Acquisition Time
FA: Flip Angle
TSE: Turbo-Spin Echo
MT: Magnetization transfer
FS: FreeSurfer
MNI: Montreal Neurological Institute
ROI: Region of interest
PVC: Partial volume correction
DVR: Distribution volume ratio
FLR: Frontal, lateral temporal and retrosplenial cortices
SUVr: Standardized uptake value ratio
IT: Inferior temporal cortex
IQR: Interquartile range
CT: Cortical thickness
MCI: Mild cognitive impairment

## References

[1] Jack CR, Bennett DA, Blennow K, Carrillo MC, Dunn B, Haeberlein SB (2018). NIA-AA Research Framework: Toward a biological definition of Alzheimer's disease. Alzheimers Dement.

[2] Lane CA, Hardy J, Schott JM (2018). Alzheimer's disease. Eur J Neurol.

[3] Braak H, Thal DR, Ghebremedhin E, Del Tredici K (2011). Stages of the pathologic process in Alzheimer disease: age categories from 1 to 100 years. J Neuropathol Exp Neurol.

[4] Samuels ER, Szabadi E (2008). Functional neuroanatomy of the noradrenergic locus coeruleus: its roles in the regulation of arousal and autonomic function part I: principles of functional organisation. Curr Neuropharmacol.

[5] Pooler AM, Polydoro M, Maury EA, Nicholls SB, Reddy SM, Wegmann S (2015). Amyloid accelerates tau propagation and toxicity in a model of early Alzheimer's disease. Acta Neuropathol Commun.

[6] Jack CR, Wiste HJ, Botha H, Weigand SD, Therneau TM, Knopman DS (2019). The bivariate distribution of amyloid-β and tau: relationship with established neurocognitive clinical syndromes. Brain.

[7] Theofilas P, Ehrenberg AJ, Dunlop S, Di Lorenzo Alho AT, Nguy A, Leite REP (2017). Locus coeruleus volume and cell population changes during Alzheimer's disease progression: A stereological study in human postmortem brains with potential implication for early-stage biomarker discovery. Alzheimers Dement.

[8] Gilvesy A, Husen E, Magloczky Z, Mihaly O, Hortobágyi T, Kanatani S (2022). Spatiotemporal characterization of cellular tau pathology in the human locus coeruleus-pericoerulear complex by three-dimensional imaging. Acta Neuropathol.

[9] Rorabaugh JM, Chalermpalanupap T, Botz-Zapp CA, Fu VM, Lembeck NA, Cohen RM (2017). Chemogenetic locus coeruleus activation restores reversal learning in a rat model of Alzheimer's disease. Brain.

[10] La Joie R, Visani AV, Baker SL, Brown JA, Bourakova V, Cha J, et al. Prospective longitudinal atrophy in Alzheimer's disease correlates with the intensity and topography of baseline tau-PET. Sci Transl Med. 2020;12(524):eaau5732. 10.1126/scitranslmed.aau5732 PMC7035952 31894103

[11] Robertson IH (2013). A noradrenergic theory of cognitive reserve: implications for Alzheimer's disease. Neurobiol Aging.

[12] Mather M, Harley CW (2016). The Locus Coeruleus: Essential for Maintaining Cognitive Function and the Aging Brain. Trends Cogn Sci.

[13] Bachman SL, Dahl MJ, Werkle-Bergner M, Duzel S, Forlim CG, Lindenberger U (2021). Locus coeruleus MRI contrast is associated with cortical thickness in older adults. Neurobiol Aging.

[14] Elman JA, Puckett OK, Hagler DJ, Pearce RC, Fennema-Notestine C, Hatton SN, Lyons MJ, McEvoy LK, Panizzon MS, Reas ET, Dale AM, Franz CE, Kremen WS. Associations Between MRI-Assessed Locus Coeruleus Integrity and Cortical Gray Matter Microstructure. Cereb Cortex. 2022;32(19):4191–203. 10.1093/cercor/bhab475 PMC9528780 34969072

[15] Dagley A, LaPoint M, Huijbers W, Hedden T, McLaren DG, Chatwal JP (2017). Harvard Aging Brain Study: Dataset and accessibility. Neuroimage.

[16] Morris J (1993). The clinical dementia rating (CDR): current version and scoring rules. Neurology.

[17] Yesavage JA, Sheikh JI. 9/Geriatric depression scale (GDS) recent evidence and development of a shorter version. Clin Gerontol. 1986;5(1-2):165–73.

[18] Folstein MF, Folstein SE, McHugh PR (1975). “Mini-mental state”: a practical method for grading the cognitive state of patients for the clinician. J Psychiatr Res.

[19] Powel J. Wechsler memory scale-revised: David A. Wechsler. New York: The Psychological Corporation. Harcourt Brace Jovanovich, Inc, 1987. 150 pp. Arch Clin Neuropsychol. 1988;3(4):397–403.

[20] The Belmont Report (2014). Ethical principles and guidelines for the protection of human subjects of research. J Am Coll Dent.

[21] Dale AM, Fischl B, Sereno MI (1999). Cortical Surface-Based Analysis: I. Segmentation and Surface Reconstruction. NeuroImage (Orlando, Fla).

[22] Jacobs HIL, Becker JA, Kwong K, Engels-Dominguez E, Prokopiou PC, Papp KV (2021). In vivo and neuropathology data support locus coeruleus integrity as indicator of Alzheimer's disease pathology and cognitive decline. Science Translational Medicine..

[23] Keren NI, Taheri S, Vazey EM, Morgan PS, Granholm AC, Aston-Jones GS (2015). Histologic validation of locus coeruleus MRI contrast in post-mortem tissue. Neuroimage.

[24] Ohm TG, Busch C, Bohl J (1997). Unbiased estimation of neuronal numbers in the human nucleus coeruleus during aging. Neurobiol Aging.

[25] German DC, Walker BS, Manaye K, Smith WK, Woodward DJ, North AJ (1988). The human locus coeruleus: computer reconstruction of cellular distribution. J Neurosci.

[26] Fernandes P, Regala J, Correia F, Goncalves-Ferreira AJ (2012). The human locus coeruleus 3-D stereotactic anatomy. Surg Radiol Anat.

[27] Braak H, Del Tredici K. Neuroanatomy and pathology of sporadic Alzheimer's disease. Adv Anat Embryol Cell Biol. 2015;215:1–162. 25920101

[28] Desikan RS, Ségonne F, Fischl B, Quinn BT, Dickerson BC, Blacker D (2006). An automated labeling system for subdividing the human cerebral cortex on MRI scans into gyral based regions of interest. NeuroImage (Orlando, Fla).

[29] Johnson KA, Schultz A, Betensky RA, Becker JA, Sepulcre J, Rentz D (2016). Tau positron emission tomographic imaging in aging and early Alzheimer disease. Ann Neurol.

[30] Becker JA, Hedden T, Carmasin J, Maye J, Rentz DM, Putcha D (2011). Amyloid- Delta b associated cortical thinning in clinically normal elderly. Ann Neurol.

[31] Greve DN, Svarer C, Fisher PM, Feng L, Hansen AE, Baare W (2014). Cortical surface-based analysis reduces bias and variance in kinetic modeling of brain PET data. NeuroImage (Orlando, Fla).

[32] Jacobs HIL, Augustinack JC, Schultz AP, Hanseeuw BJ, Locascio J, Amariglio RE (2020). The presubiculum links incipient amyloid and tau pathology to memory function in older persons. Neurology.

[33] Maass A, Landau S, Baker SL, Horng A, Lockhart SN, La Joie R (2017). Comparison of multiple tau-PET measures as biomarkers in aging and Alzheimer's disease. Neuroimage.

[34] Rodriguez-Vieitez E, Montal V, Sepulcre J, Lois C, Hanseeuw B, Vilaplana E (2021). Association of cortical microstructure with amyloid-β and tau: impact on cognitive decline, neurodegeneration, and clinical progression in older adults. Mol Psychiatry.

[35] Buckley RF, Hanseeuw B, Schultz AP, Vannini P, Aghjayan SL, Properzi MJ (2017). Region-Specific Association of Subjective Cognitive Decline With Tauopathy Independent of Global β-Amyloid Burden. JAMA Neurol.

[36] Therriault J, Pascoal TA, Lussier FZ, Tissot C, Chamoun M, Bezgin G (2022). Biomarker modeling of Alzheimer's disease using PET-based Braak staging. Nat Aging.

[37] Cohen J. Statistical Power Analysis for the Behavioral Sciences. 2nd ed. United States of America: Routledge; 1988;1–567.

[38] Betts MJ, Kirilina E, Otaduy MCG, Ivanov D, Acosta-Cabronero J, Callaghan MF (2019). Locus coeruleus imaging as a biomarker for noradrenergic dysfunction in neurodegenerative diseases. Brain.

[39] Cassidy CM, Therriault J, Pascoal TA, Cheung V, Savard M, Tuominen L (2022). Association of locus coeruleus integrity with Braak stage and neuropsychiatric symptom severity in Alzheimer's disease. Neuropsychopharmacology.

[40] Dahl MJ, Mather M, Werkle-Bergner M, Kennedy BL, Guzman S, Hurth K (2021). Locus coeruleus integrity is related to tau burden and memory loss in autosomal-dominant Alzheimer's disease. Neurobiol Aging.

[41] Weinshenker D (2018). Long Road to Ruin: Noradrenergic Dysfunction in Neurodegenerative Disease. Trends Neurosci.

[42] Raskind MA, Peskind ER, Holmes C, Goldstein DS (1999). Patterns of cerebrospinal fluid catechols support increased central noradrenergic responsiveness in aging and Alzheimer's disease. Biol Psychiatry.

[43] Sheline YI, Miller K, Bardgett ME, Csernansky JG (1998). Higher cerebrospinal fluid MHPG in subjects with dementia of the Alzheimer type. Relationship with cognitive dysfunction. Am J Geriatr Psychiatry.

[44] Szot P, White SS, Greenup JL, Leverenz JB, Peskind ER, Raskind MA (2006). Compensatory changes in the noradrenergic nervous system in the locus ceruleus and hippocampus of postmortem subjects with Alzheimer's disease and dementia with Lewy bodies. J Neurosci.

[45] Elrod R, Peskind ER, DiGiacomo L, Brodkin KI, Veith RC, Raskind MA (1997). Effects of Alzheimer's disease severity on cerebrospinal fluid norepinephrine concentration. Am J Psychiatry.

[46] Hoogendijk WJG, Feenstra MGP, Botterblom MHA, Gilhuis J, Sommer IEC, Kamphorst W (1999). Increased Activity of Surviving Locus Ceruleus Neurons in Alzheimers Disease. Ann Neurol.

[47] Theofilas P, Dunlop S, Heinsen H, Grinberg LT (2015). Turning on the Light Within: Subcortical Nuclei of the Isodentritic Core and their Role in Alzheimer's Disease Pathogenesis. Journal of Alzheimer’s Disease.

[48] Palmer AM, Wilcock GK, Esiri MM, Francis PT, Bowen DM (1987). Monoaminergic innervation of the frontal and temporal lobes in Alzheimer's disease. Brain Res.

[49] Tejani-Butt SM, Yang J, Zaffar H (1993). Norepinephrine transporter sites are decreased in the locus coeruleus in Alzheimer's disease. Brain Res.

[50] Matthews KL, Chen CP, Esiri MM, Keene J, Minger SL, Francis PT (2002). Noradrenergic changes, aggressive behavior, and cognition in patients with dementia. Biol Psychiatry.

[51] Raskind MA, Peskind ER (1994). Neurobiologic bases of noncognitive behavioral problems in Alzheimer disease. Alzheimer Dis Assoc Disord.

[52] Adolfsson R, Gottfries CG, Roos BE, Winblad B (1979). Changes in the brain catecholamines in patients with dementia of Alzheimer type. Br J Psychiatry.

[53] Riphagen JM, van Egroo M, Jacobs HIL (2021). Elevated Norepinephrine Metabolism Gauges Alzheimer's Disease-Related Pathology and Memory Decline. J Alzheimers Dis.

[54] Jacobs HIL, Riphagen JM, Ramakers I, Verhey FRJ (2021). Alzheimer's disease pathology: pathways between central norepinephrine activity, memory, and neuropsychiatric symptoms. Mol Psychiatry.

[55] Kalinin S, Gavrilyuk V, Polak PE, Vasser R, Zhao J, Heneka MT (2007). Noradrenaline deficiency in brain increases beta-amyloid plaque burden in an animal model of Alzheimer's disease. Neurobiol Aging.

[56] Jardanhazi-Kurutz D, Kummer MP, Terwel D, Vogel K, Dyrks T, Thiele A (2010). Induced LC degeneration in APP/PS1 transgenic mice accelerates early cerebral amyloidosis and cognitive deficits. Neurochem Int.

[57] Heneka MT, Nadrigny F, Regen T, Martinez-Hernandez A, Dumitrescu-Ozimek L, Terwel D (2010). Locus ceruleus controls Alzheimer's disease pathology by modulating microglial functions through norepinephrine. Proc Natl Acad Sci U S A.

[58] Heneka MT, Ramanathan M, Jacobs AH, Dumitrescu-Ozimek L, Bilkei-Gorzo A, Debeir T (2006). Locus ceruleus degeneration promotes Alzheimer pathogenesis in amyloid precursor protein 23 transgenic mice. J Neurosci.

[59] Mercan D, Heneka MT. The Contribution of the Locus Coeruleus-Noradrenaline System Degeneration during the Progression of Alzheimer's Disease. Biology (Basel). 2022;11(12):1822. 10.3390/biology11121822 PMC9775634 36552331

[60] Chalermpalanupap T, Schroeder JP, Rorabaugh JM, Liles LC, Lah JJ, Levey AI (2018). Locus Coeruleus Ablation Exacerbates Cognitive Deficits, Neuropathology, and Lethality in P301S Tau Transgenic Mice. J Neurosci.

[61] van Hooren RWE, Verhey FRJ, Ramakers I, Jansen WJ, Jacobs HIL (2021). Elevated norepinephrine metabolism is linked to cortical thickness in the context of Alzheimer's disease pathology. Neurobiol Aging.

[62] Levinson S, Miller M, Iftekhar A, Justo M, Arriola D, Wei W (2022). A structural connectivity atlas of limbic brainstem nuclei. Front Neuroimaging.

[63] Fathy YY, Hoogers SE, Berendse HW, van der Werf YD, Visser PJ, de Jong FJ (2020). Differential insular cortex sub-regional atrophy in neurodegenerative diseases: a systematic review and meta-analysis. Brain Imaging Behav.

[64] Foundas AL, Leonard CM, Mahoney SM, Agee OF, Heilman KM (1997). Atrophy of the Hippocampus, Parietal Cortex, and Insula in Alzheimer's Disease: A Volumetric Magnetic Resonance Imaging Study. Cogn Behav Neurol.

[65] Rombouts SA, Barkhof F, Witter MP, Scheltens P (2000). Unbiased whole-brain analysis of gray matter loss in Alzheimer's disease. Neurosci Lett.

[66] Karas G, Scheltens P, Rombouts SA, Visser PJ, van Schijndel RA, Fox NC (2004). Global and local gray matter loss in mild cognitive impairment and Alzheimer's disease. Neuroimage.

[67] Augustine JR (1996). Circuitry and functional aspects of the insular lobe in primates including humans. Brain Res Rev.

[68] Zhou J, Greicius MD, Gennatas ED, Growdon ME, Jang JY, Rabinovici GD (2010). Divergent network connectivity changes in behavioural variant frontotemporal dementia and Alzheimer's disease. Brain.

[69] Seeley WW (2019). The salience network: a neural system for perceiving and responding to homeostatic demands. J Neurosci.

[70] Chen S, Xu W, Xue C, Hu G, Ma W, Qi W (2020). Voxelwise meta-analysis of gray matter abnormalities in mild cognitive impairment and subjective cognitive decline using activation likelihood estimation. J Alzheimers Dis.

[71] Gordon BA, McCullough A, Mishra S, Blazey TM, Su Y, Christensen J (2018). Cross-sectional and longitudinal atrophy is preferentially associated with tau rather than amyloid β positron emission tomography pathology. Alzheimer's & Dementia: Diagnosis, Assessment & Disease Monitoring.

[72] Tondelli M, Wilcock GK, Nichelli P, De Jager CA, Jenkinson M, Zamboni G (2012). Structural MRI changes detectable up to ten years before clinical Alzheimer's disease. Neurobiol Aging..

[73] Trujillo P, Summers PE, Ferrari E, Zucca FA, Sturini M, Mainardi LT (2017). Contrast mechanisms associated with neuromelanin-MRI. Magn Reson Med.

[74] Watanabe T, Tan Z, Wang X, Martinez-Hernandez A, Frahm J (2019). Magnetic resonance imaging of noradrenergic neurons. Brain Struct Funct.

[75] Priovoulos N, van Boxel SCJ, Jacobs HIL, Poser BA, Uludag K, Verhey FRJ (2020). Unraveling the contributions to the neuromelanin-MRI contrast. Brain Struct Funct.

[76] Engels-Domínguez N, Koops EA, Prokopiou PC, Van Egroo M, Schneider C, Riphagen JM (2023). State-of-the-art imaging of neuromodulatory subcortical systems in aging and Alzheimer's disease: Challenges and opportunities. Neurosci Biobehav Rev.

[77] Crary JF, Trojanowski JQ, Schneider JA, Abisambra JF, Abner EL, Alafuzoff I (2014). Primary age-related tauopathy (PART): a common pathology associated with human aging. Acta Neuropathol.

