# Supplementary material for: Lower in vivo locus coeruleus integrity is associated with lower cortical thickness in older individuals with elevated Alzheimer’s pathology: a cohort study
Source: Alzheimers Res Ther. 2024 Jun 17;16:129. doi: 10.1186/s13195-024-01500-0 (PMC11181564; doi:10.1186/s13195-024-01500-0)
Supplement: Supplementary file 1 — Supplementary Material 1. [file 13195_2024_1500_MOESM1_ESM.docx]

**SUPPLEMENTARY MATERIAL**

**Lower *in vivo* locus coeruleus integrity is associated with lower cortical thickness in older individuals with elevated Alzheimer’s pathology: a cohort study**

**Authors:** Nina Engels-Domínguez^1,2^, Elouise A. Koops^1^, Stephanie Hsieh^1^, Emma E. Wiklund^1^, Aaron P. Schultz^1,3^, Joost M. Riphagen^1^, Prokopis C. Prokopiou^1^, Bernard J. Hanseeuw^3,4,5^, Dorene M. Rentz^3,6^, Reisa A. Sperling^3,6^, Keith A. Johnson^3,4,6^, Heidi I.L. Jacobs^1,2^

**Affiliations:**

^1^ The Athinoula A. Martinos Center for Biomedical Imaging, Department of Radiology, Massachusetts General Hospital, Harvard Medical School, Boston, MA, USA.

^2^ Faculty of Health, Medicine and Life Sciences, School for Mental Health and Neuroscience, Alzheimer Centre Limburg, Maastricht University, Maastricht, The Netherlands.

^3^ Department of Neurology, Massachusetts General Hospital, Harvard Medical School, Boston, MA, USA.

^4^ Gordon Center for Medical Imaging, Department of Radiology, Massachusetts General Hospital, Harvard Medical School, Boston, MA, USA.

^5^ Department of Neurology, Cliniques Universitaires Saint-Luc, Brussels, Belgium.

^6^ Center for Alzheimer Research and Treatment, Department of Neurology, Brigham and Women’s Hospital, Harvard Medical School, Boston, MA, USA.

* To whom correspondence should be addressed: Dr. Heidi Jacobs, PhD, Department of Radiology, Massachusetts General Hospital/Harvard Medical School, Boston MA 02114, USA, Email: hjacobs@mgh.harvard.edu

| **Characteristics of CDR = 0 participants sorted on PiB status** | | | | |
| --- | --- | --- | --- | --- |
|  | Low  beta-amyloid | Elevated  beta-amyloid | P-value |  |
| n | 106 | 43 |  |  |
| Age | 71.27 (9.42) | 78.91 (7.96) | **< 0.001 **** |  |
| Sex, No. (%) = M | 43 (40.6) | 19 (44.2) | 0.824 |  |
| Years of Education | 16.29 (2.89) | 16.49 (2.94) | 0.710 |  |
| Race, No. (%) |  |  | 0.828 |  |
| AS | 3 (2.8) | 0 (0.0) |  |  |
| B | 12 (11.3) | 4 (9.3) |  |  |
| NH | 16 (15.1) | 6 (14.0) |  |  |
| Other | 1 (0.9) | 1 (2.3) |  |  |
| Unknown | 4 (3.8) | 1 (2.3) |  |  |
| W | 70 (66.0) | 31 (72.1) |  |  |
| APOE-ε4 Carrier Status, No. (%) = ε4+ | 12 (11.8) | 23 (54.8) | **< 0.001 **** |  |
| LC MRI signal intensity | 1.34 (0.05) | 1.30 (0.03) | **< 0.001 **** |  |
| IT FTP values (SUVr, PVC) | 1.41 (0.16) | 1.60 (0.31) | **< 0.001 **** |  |
| EC FTP values (SUVr, PVC) | 1.27 (0.25) | 1.48 (0.34) | **< 0.001 **** |  |
| FLR-PiB values (DVR, PVC) | 1.19 (0.06) | 1.90 (0.47) | **< 0.001 **** |  |
| Clinical Dementia Rating, No. (%) = 0 | 106 (100.0) | 43 (100.0) |  |  |
| MMSE Score | 29.04 (1.25) | 28.53 (1.50) | **0.038 *** |  |
| **Supplemental Table 1.** **Characteristics of CDR = 0 participants sorted on PiB status.** Data is presented as numbers and (percentages) or means and (standard deviations). Chi-square tests and Kruskal tests were conducted to reveal group differences for categorical and non-normal continuous variables, respectively. Missing data for APOE- ε4 Carrier Status (n = 5). Abbreviations: No, number; M, male; AS, Asian; B, Black or African American; NH, Native Hawaiian or other Pacific Islander; W, White; LC, locus coeruleus; IT, inferior temporal; FTP, [^18^F]-flortaucipir; SUVr, standardized uptake value ratio; PVC, partial-volume corrected; EC, entorhinal cortex; FLR, frontal, laterotemporal and retrosplenial cortices; PiB, [^11^C]-Pittsburgh Compound-B; DVR, distribution volume ratio; MMSE, Mini-Mental State Examination. ***P** < .05; ****P** < .001. | | | | |

**Lower LC MRI signal intensity is associated with lower mediolateral temporal cortical thickness**

| **Cortical thickness region** | ***B*** | **Lower 95% CI** | **Upper 95% CI** | ***P*-value** | ***P_FDR_*-value** |
| --- | --- | --- | --- | --- | --- |
| bankssts | 0.552 | 0.031 | 1.051 | **0.039** | 0.064 ^ |
| caudal anterior cingulate | -0.083 | -0.781 | 0.630 | 0.802 | 0.887 |
| caudal middle frontal | 0.242 | -0.172 | 0.666 | 0.256 | 0.427 |
| cuneus | -0.055 | -0.416 | 0.294 | 0.754 | 0.754 |
| entorhinal | 2.055 | 0.879 | 3.253 | **0.000** | **0.000** |
| fusiform | 0.806 | 0.392 | 1.220 | **0.000** | **0.000** |
| inferior parietal | 0.175 | -0.200 | 0.555 | 0.350 | 0.438 |
| inferior temporal | 0.698 | 0.258 | 1.110 | **0.002** | **0.005** |
| isthmus cingulate | 0.579 | 0.008 | 1.147 | **0.048** | 0.079 |
| lateral occipital | 0.212 | -0.149 | 0.574 | 0.237 | 0.394 |
| lateral orbitofrontal | 0.322 | -0.033 | 0.674 | 0.075 | 0.124 |
| lingual | 0.583 | 0.282 | 0.908 | **0.000** | **0.000** |
| medial orbitofrontal | 0.178 | -0.209 | 0.601 | 0.394 | 0.909 |
| middle temporal | 0.634 | 0.209 | 1.057 | **0.003** | **0.005** |
| parahippocampal | 1.081 | 0.221 | 1.930 | **0.013** | **0.031** |
| paracentral | 0.659 | 0.185 | 1.124 | **0.014** | **0.035** |
| pars opercularis | 0.345 | -0.033 | 0.722 | 0.070 | 0.087 |
| pars orbitalis | 0.196 | -0.291 | 0.665 | 0.424 | 0.707 |
| pars triangularis | 0.327 | -0.071 | 0.727 | 0.115 | 0.286 |
| pericalcarine | 0.176 | -0.186 | 0.563 | 0.325 | 0.651 |
| postcentral | 0.391 | -0.022 | 0.818 | 0.069 ^ | 0.173 |
| posterior cingulate | 0.280 | -0.217 | 0.774 | 0.267 | 0.334 |
| precentral | 0.517 | 0.048 | 1.007 | **0.020** | **0.033** |
| precuneus | 0.195 | -0.222 | 0.585 | 0.350 | 0.583 |
| rostral anterior cingulate | 0.253 | -0.370 | 0.838 | 0.416 | 0.575 |
| rostral middle frontal | 0.274 | -0.057 | 0.618 | 0.123 | 0.223 |
| superior frontal | 0.294 | -0.117 | 0.695 | 0.161 | 0.268 |
| superior parietal | 0.194 | -0.253 | 0.630 | 0.392 | 0.653 |
| superior temporal | 0.773 | 0.256 | 1.278 | **0.002** | **0.003** |
| supramarginal | 0.355 | -0.053 | 0.739 | 0.085 | 0.141 |
| frontal pole | 0.423 | -0.240 | 1.135 | 0.214 | 0.356 |
| temporal pole | 1.470 | 0.482 | 2.410 | **0.004** | **0.006** |
| transverse temporal | 0.743 | 0.044 | 1.402 | **0.041** | 0.068 ^ |
| insula | 0.760 | 0.311 | 1.205 | **0.003** | **0.006** |

**Supplemental Table 2. Associations between measures of cortical thickness and LC MRI signal intensity for the entire sample, corrected for age, sex, and years of education.** Analyses were performed using bootstrapped linear regressions with cortical thickness regions as the dependent variable, LC MRI signal intensity as the independent variable, and age, sex and years of education as covariates (n = 165). Reported regression coefficients are unstandardized, and P-values have been corrected for multiple comparisons using FDR. Significant P-values have been indicated in bold. P-values at trend level are indicated with ^. Lower LC MRI signal intensity is associated with lower entorhinal, fusiform, inferior temporal, lingual, middle temporal, parahippocampal, paracentral, precentral, superior temporal, temporal polar and insular cortical thickness. Abbreviations: CI, confidence interval; FDR, False Discovery Rate; Bankssts, banks of superior temporal sulcus.

| **Cortical thickness region** | ***B*** | **Lower 95% CI** | **Upper 95% CI** | ***P*-value** | ***P_FDR_*-value** |
| --- | --- | --- | --- | --- | --- |
| bankssts | 0.366 | -0.131 | 0.866 | 0.128 | 0.255 |
| caudal anterior cingulate | -0.360 | -1.025 | 0.349 | 0.330 | 0.330 |
| caudal middle frontal | 0.113 | -0.296 | 0.547 | 0.582 | 0.597 |
| cuneus | -0.099 | -0.481 | 0.278 | 0.589 | 0.621 |
| entorhinal | 1.922 | 0.791 | 3.014 | **0.002** | **0.004** |
| fusiform | 0.576 | 0.162 | 0.978 | **0.007** | **0.010** |
| inferior parietal | 0.005 | -0.368 | 0.377 | 0.960 | 0.960 |
| inferior temporal | 0.575 | 0.163 | 0.989 | **0.008** | **0.022** |
| isthmus cingulate | 0.397 | -0.185 | 0.962 | 0.179 | 0.215 |
| lateral occipital | 0.068 | -0.290 | 0.416 | 0.706 | 0.847 |
| lateral orbitofrontal | 0.263 | -0.094 | 0.608 | 0.154 | 0.266 |
| lingual | 0.493 | 0.163 | 0.814 | **0.002** | **0.006** |
| medial orbitofrontal | 0.064 | -0.348 | 0.478 | 0.754 | 0.987 |
| middle temporal | 0.474 | 0.047 | 0.874 | **0.023** | **0.034** |
| parahippocampal | 0.773 | -0.064 | 1.644 | 0.089 | 0.133 |
| paracentral | 0.547 | 0.064 | 1.020 | **0.030** | 0.089 |
| pars opercularis | 0.262 | -0.119 | 0.632 | 0.186 | 0.223 |
| pars orbitalis | 0.127 | -0.374 | 0.627 | 0.621 | 0.905 |
| pars triangularis | 0.246 | -0.154 | 0.618 | 0.208 | 0.335 |
| pericalcarine | 0.093 | -0.269 | 0.469 | 0.594 | 0.712 |
| postcentral | 0.301 | -0.119 | 0.712 | 0.149 | 0.298 |
| posterior cingulate | 0.126 | -0.344 | 0.630 | 0.603 | 0.603 |
| precentral | 0.400 | -0.067 | 0.896 | 0.102 | 0.152 |
| precuneus | 0.017 | -0.376 | 0.415 | 0.911 | 0.911 |
| rostral anterior cingulate | 0.016 | -0.602 | 0.636 | 0.928 | 0.928 |
| rostral middle frontal | 0.157 | -0.175 | 0.506 | 0.340 | 0.340 |
| superior frontal | 0.143 | -0.253 | 0.521 | 0.476 | 0.571 |
| superior parietal | 0.037 | -0.423 | 0.489 | 0.846 | 0.846 |
| superior temporal | 0.642 | 0.150 | 1.153 | **0.017** | **0.034** |
| supramarginal | 0.191 | -0.203 | 0.580 | 0.316 | 0.379 |
| frontal pole | 0.268 | -0.393 | 0.998 | 0.458 | 0.576 |
| temporal pole | 1.405 | 0.383 | 2.394 | **0.010** | **0.019** |
| transverse temporal | 0.648 | -0.060 | 1.340 | 0.073 | 0.146 |
| insula | 0.664 | 0.190 | 1.129 | **0.005** | **0.015** |

**Supplemental Table 3. Associations between measures of cortical thickness and LC MRI signal intensity for the entire sample, corrected for neocortical PiB, age, sex, and years of education.** Analyses were performed using bootstrapped linear regressions with cortical thickness regions as the dependent variable, LC MRI signal intensity as the independent variable, and neocortical PiB, age, sex and years of education as covariates (n = 165). Reported regression coefficients are unstandardized, and P-values have been corrected for multiple comparisons using FDR. Significant P-values have been indicated in bold. Significant P-values of regions in models with non-PVC data have been outlined by a blue box. Lower LC MRI signal intensity is associated with lower entorhinal, fusiform, inferior temporal, lingual, middle temporal, superior temporal, temporal polar and insular cortical thickness. Abbreviations: CI, confidence interval; FDR, False Discovery Rate; Bankssts, banks of superior temporal sulcus.

| **Cortical thickness region** | ***B*** | **Lower 95% CI** | **Upper 95% CI** | ***P*-value** | ***P_FDR_*-value** |
| --- | --- | --- | --- | --- | --- |
| bankssts | 0.458 | -0.074 | 0.980 | 0.089 | 0.201 |
| caudal anterior cingulate | -0.210 | -0.952 | 0.501 | 0.560 | 0.651 |
| caudal middle frontal | 0.119 | -0.340 | 0.550 | 0.579 | 0.645 |
| cuneus | -0.095 | -0.497 | 0.299 | 0.643 | 0.643 |
| entorhinal | 2.050 | 0.847 | 3.265 | **0.003** | **0.005** |
| fusiform | 0.584 | 0.127 | 1.029 | **0.012** | **0.017** |
| inferior parietal | -0.017 | -0.413 | 0.358 | 0.963 | 0.963 |
| inferior temporal | 0.579 | 0.138 | 1.003 | **0.006** | **0.017** |
| isthmus cingulate | 0.477 | -0.113 | 1.059 | 0.124 | 0.194 |
| lateral occipital | 0.105 | -0.253 | 0.472 | 0.591 | 0.756 |
| lateral orbitofrontal | 0.234 | -0.141 | 0.594 | 0.211 | 0.317 |
| lingual | 0.490 | 0.167 | 0.802 | **0.003** | **0.008** |
| medial orbitofrontal | 0.066 | -0.361 | 0.481 | 0.761 | 0.995 |
| middle temporal | 0.467 | 0.047 | 0.885 | **0.032** | **0.048** |
| parahippocampal | 0.906 | 0.002 | 1.762 | **0.049** | 0.098 |
| paracentral | 0.590 | 0.109 | 1.078 | **0.021** | 0.063 ^ |
| pars opercularis | 0.255 | -0.112 | 0.642 | 0.177 | 0.212 |
| pars orbitalis | 0.067 | -0.430 | 0.540 | 0.779 | 0.986 |
| pars triangularis | 0.233 | -0.182 | 0.643 | 0.250 | 0.375 |
| pericalcarine | -0.011 | -0.367 | 0.365 | 0.955 | 0.955 |
| postcentral | 0.316 | -0.145 | 0.767 | 0.157 | 0.470 |
| posterior cingulate | 0.157 | -0.378 | 0.636 | 0.522 | 0.522 |
| precentral | 0.451 | -0.065 | 0.967 | 0.082 | 0.163 |
| precuneus | -0.002 | -0.422 | 0.396 | 0.991 | 0.991 |
| rostral anterior cingulate | 0.023 | -0.600 | 0.629 | 0.956 | 0.956 |
| rostral middle frontal | 0.152 | -0.197 | 0.496 | 0.393 | 0.393 |
| superior frontal | 0.137 | -0.282 | 0.532 | 0.511 | 0.613 |
| superior parietal | 0.007 | -0.418 | 0.456 | 0.989 | 0.989 |
| superior temporal | 0.639 | 0.105 | 1.153 | **0.021** | **0.041** |
| supramarginal | 0.184 | -0.223 | 0.589 | 0.347 | 0.416 |
| frontal pole | 0.197 | -0.509 | 0.901 | 0.576 | 0.691 |
| temporal pole | 1.408 | 0.402 | 2.431 | **0.005** | **0.009** |
| transverse temporal | 0.681 | -0.042 | 1.421 | 0.072 | 0.144 |
| insula | 0.684 | 0.209 | 1.151 | **0.007** | **0.021** |

**Supplemental Table 4. Associations between measures of cortical thickness and LC MRI signal intensity for the entire sample, corrected for neocortical PiB status, age, sex, and years of education.** Analyses were performed using bootstrapped linear regressions with cortical thickness regions as the dependent variable, LC MRI signal intensity as the independent variable, and neocortical PiB status, age, sex and years of education as covariates (n = 165). Reported regression coefficients are unstandardized, and P-values have been corrected for multiple comparisons using FDR. Significant P-values have been indicated in bold. P-values at trend level are indicated with ^. Significant P-values of regions in models with non-PVC data have been outlined by a blue box. Lower LC MRI signal intensity is associated with lower entorhinal, fusiform, inferior temporal, lingual, middle temporal, superior temporal, temporal polar and insular cortical thickness. Abbreviations: CI, confidence interval; FDR, False Discovery Rate; Bankssts, banks of superior temporal sulcus.

| Cortical thickness region | *B* | Lower 95% CI | Upper 95% CI | *P*-value | *P_FDR_*-value |
| --- | --- | --- | --- | --- | --- |
| bankssts | 0.393 | -0.123 | 0.939 | 0.136 | 0.226 |
| caudal anterior cingulate | -0.330 | -1.136 | 0.428 | 0.379 | 0.504 |
| caudal middle frontal | -0.006 | -0.432 | 0.429 | 0.920 | 0.920 |
| cuneus | -0.243 | -0.655 | 0.193 | 0.262 | 0.436 |
| entorhinal | 1.195 | -0.018 | 2.371 | 0.054 ^ | 0.068 ^ |
| fusiform | 0.265 | -0.131 | 0.648 | 0.197 | 0.328 |
| inferior parietal | -0.186 | -0.613 | 0.199 | 0.367 | 0.504 |
| inferior temporal | 0.329 | -0.098 | 0.729 | 0.126 | 0.314 |
| isthmus cingulate | 0.253 | -0.423 | 0.932 | 0.447 | 0.558 |
| lateral occipital | -0.084 | -0.457 | 0.271 | 0.635 | 0.635 |
| lateral orbitofrontal | 0.044 | -0.320 | 0.430 | 0.819 | 0.989 |
| lingual | 0.346 | -0.037 | 0.694 | 0.067 ^ | 0.168 |
| medial orbitofrontal | 0.032 | -0.435 | 0.515 | 0.907 | 0.999 |
| middle temporal | 0.191 | -0.233 | 0.604 | 0.353 | 0.588 |
| parahippocampal | 0.488 | -0.326 | 1.338 | 0.271 | 0.271 |
| paracentral | 0.269 | -0.209 | 0.747 | 0.269 | 0.448 |
| pars opercularis | 0.108 | -0.287 | 0.516 | 0.645 | 0.806 |
| pars orbitalis | -0.058 | -0.534 | 0.481 | 0.817 | 0.976 |
| pars triangularis | 0.051 | -0.357 | 0.468 | 0.808 | 0.861 |
| pericalcarine | -0.170 | -0.590 | 0.261 | 0.392 | 0.392 |
| postcentral | 0.208 | -0.311 | 0.698 | 0.381 | 0.634 |
| posterior cingulate | 0.133 | -0.431 | 0.677 | 0.671 | 0.671 |
| precentral | 0.234 | -0.293 | 0.698 | 0.379 | 0.593 |
| precuneus | -0.134 | -0.535 | 0.244 | 0.491 | 0.613 |
| rostral anterior cingulate | -0.222 | -0.918 | 0.461 | 0.524 | 0.662 |
| rostral middle frontal | 0.012 | -0.318 | 0.355 | 0.969 | 0.969 |
| superior frontal | 0.027 | -0.362 | 0.416 | 0.877 | 0.877 |
| superior parietal | -0.071 | -0.518 | 0.386 | 0.777 | 0.965 |
| superior temporal | 0.337 | -0.206 | 0.831 | 0.207 | 0.345 |
| supramarginal | -0.004 | -0.467 | 0.441 | 0.999 | 0.999 |
| frontal pole | 0.182 | -0.552 | 0.912 | 0.641 | 0.648 |
| temporal pole | 0.963 | -0.074 | 2.015 | 0.082 | 0.136 |
| transverse temporal | 0.326 | -0.424 | 1.091 | 0.405 | 0.506 |
| insula | 0.339 | -0.162 | 0.808 | 0.177 | 0.295 |

**Supplemental Table 5. Associations between measures of cortical thickness and LC MRI signal intensity for the low beta-amyloid sample, corrected for age, sex, and years of education.** Analyses were performed using bootstrapped linear regressions with cortical thickness regions as the dependent variable, LC MRI signal intensity as the independent variable, and age, sex and years of education as covariates (n = 112). Reported regression coefficients are unstandardized, and P-values have been corrected for multiple comparisons using FDR. P-values at trend level are indicated with ^. Significant P-values of regions in models with non-PVC data have been outlined by a blue box. In low beta-amyloid individuals, LC MRI signal intensity is not associated with cortical thickness. Abbreviations: CI, confidence interval; FDR, False Discovery Rate; Bankssts, banks of superior temporal sulcus.

| **Cortical thickness region** | ***B*** | **Lower 95% CI** | **Upper 95% CI** | ***P*-value** | ***P_FDR_*-value** |
| --- | --- | --- | --- | --- | --- |
| bankssts | 0.412 | -0.081 | 0.906 | 0.093 | 0.231 |
| caudal anterior cingulate | -0.236 | -0.997 | 0.532 | 0.568 | 0.710 |
| caudal middle frontal | 0.008 | -0.404 | 0.430 | 0.938 | 0.938 |
| cuneus | -0.091 | -0.468 | 0.254 | 0.611 | 0.720 |
| entorhinal | 1.490 | 0.421 | 2.537 | **0.010** | **0.013** |
| fusiform | 0.552 | 0.148 | 0.983 | **0.010** | **0.016** |
| inferior parietal | -0.017 | -0.392 | 0.354 | 0.944 | 0.944 |
| inferior temporal | 0.414 | 0.037 | 0.792 | **0.025** | 0.061 ^ |
| isthmus cingulate | 0.513 | -0.055 | 1.118 | 0.079 | 0.131 |
| lateral occipital | 0.026 | -0.307 | 0.384 | 0.867 | 0.867 |
| lateral orbitofrontal | 0.193 | -0.198 | 0.552 | 0.326 | 0.543 |
| lingual | 0.436 | 0.143 | 0.747 | **0.005** | **0.013** |
| medial orbitofrontal | 0.177 | -0.255 | 0.626 | 0.416 | 0.982 |
| middle temporal | 0.378 | 0.007 | 0.747 | **0.047** | 0.078 |
| parahippocampal | 0.582 | -0.272 | 1.458 | 0.187 | 0.234 |
| paracentral | 0.313 | -0.160 | 0.777 | 0.207 | 0.344 |
| pars opercularis | 0.248 | -0.109 | 0.609 | 0.212 | 0.348 |
| pars orbitalis | 0.152 | -0.337 | 0.652 | 0.546 | 0.892 |
| pars triangularis | 0.192 | -0.216 | 0.596 | 0.337 | 0.562 |
| pericalcarine | 0.100 | -0.251 | 0.446 | 0.614 | 0.628 |
| postcentral | 0.251 | -0.181 | 0.680 | 0.267 | 0.444 |
| posterior cingulate | 0.227 | -0.234 | 0.690 | 0.337 | 0.343 |
| precentral | 0.220 | -0.275 | 0.673 | 0.352 | 0.440 |
| precuneus | -0.029 | -0.439 | 0.367 | 0.893 | 0.893 |
| rostral anterior cingulate | 0.115 | -0.519 | 0.723 | 0.734 | 0.734 |
| rostral middle frontal | 0.147 | -0.190 | 0.477 | 0.396 | 0.396 |
| superior frontal | 0.150 | -0.286 | 0.560 | 0.483 | 0.506 |
| superior parietal | -0.019 | -0.457 | 0.411 | 0.925 | 0.971 |
| superior temporal | 0.591 | 0.089 | 1.063 | **0.025** | **0.041** |
| supramarginal | 0.178 | -0.200 | 0.576 | 0.356 | 0.356 |
| frontal pole | 0.339 | -0.375 | 1.081 | 0.340 | 0.529 |
| temporal pole | 1.298 | 0.356 | 2.244 | **0.008** | **0.013** |
| transverse temporal | 0.420 | -0.379 | 1.108 | 0.294 | 0.489 |
| insula | 0.551 | 0.083 | 1.021 | **0.021** | 0.051 ^ |

**Supplemental Table 6.** **Associations between measures of cortical thickness and LC MRI signal intensity for the CDR = 0 sample, corrected for age, sex, and years of education.** Analyses were performed using bootstrapped linear regressions with cortical thickness regions as the dependent variable, LC MRI signal intensity as the independent variable, and age, sex and years of education as covariates (n = 149). Reported regression coefficients are unstandardized, and P-values have been corrected for multiple comparisons using FDR. Significant P-values have been indicated in bold, and P-values at trend level are indicated with ^. In individuals with CDR = 0, lower LC MRI signal intensity is associated with lower entorhinal, fusiform, lingual, superior temporal and temporal polar cortical thickness. Abbreviations: CI, confidence interval; FDR, False Discovery Rate; Bankssts, banks of superior temporal sulcus.

**Lower LC MRI signal intensity is related to lower cortical thickness, particularly in individuals with elevated pathology**

| Cortical thickness region | *B* | Lower 95% CI | Upper 95% CI | *P*-value | *P_FDR_*-value |
| --- | --- | --- | --- | --- | --- |
| bankssts | 0.734 | -0.230 | 1.761 | 0.146 | 0.255 |
| caudal anterior cingulate | -0.194 | -1.592 | 1.308 | 0.772 | 0.947 |
| caudal middle frontal | 0.571 | -0.242 | 1.436 | 0.179 | 0.313 |
| cuneus | 0.197 | -0.528 | 0.992 | 0.581 | 0.669 |
| entorhinal | 4.233 | 2.345 | 6.242 | **0.000** | **0.000** |
| fusiform | 1.485 | 0.659 | 2.333 | **0.000** | **0.000** |
| inferior parietal | 1.081 | 0.369 | 1.878 | **0.002** | **0.005** |
| inferior temporal | 1.079 | 0.299 | 1.929 | **0.009** | **0.020** |
| isthmus cingulate | 1.160 | 0.009 | 2.289 | **0.049** | 0.086 |
| lateral occipital | 1.128 | 0.484 | 1.861 | **0.004** | **0.006** |
| lateral orbitofrontal | 0.588 | -0.122 | 1.327 | 0.108 | 0.252 |
| lingual | 0.912 | 0.271 | 1.570 | **0.003** | **0.007** |
| medial orbitofrontal | 0.615 | -0.281 | 1.460 | 0.142 | 0.330 |
| middle temporal | 1.287 | 0.510 | 2.190 | **0.000** | **0.000** |
| parahippocampal | 2.070 | 0.431 | 3.870 | **0.011** | **0.025** |
| paracentral | 0.801 | -0.155 | 1.809 | 0.095 | 0.222 |
| pars opercularis | 0.541 | -0.228 | 1.324 | 0.162 | 0.226 |
| pars orbitalis | -0.247 | -1.247 | 0.727 | 0.610 | 0.908 |
| pars triangularis | 0.583 | -0.199 | 1.407 | 0.147 | 0.343 |
| pericalcarine | 0.731 | -0.066 | 1.409 | 0.064 ^ | 0.148 |
| postcentral | 0.604 | -0.272 | 1.506 | 0.174 | 0.405 |
| posterior cingulate | -0.109 | -1.080 | 0.895 | 0.827 | 0.917 |
| precentral | 1.122 | 0.162 | 2.141 | **0.021** | **0.037** |
| precuneus | 0.619 | -0.150 | 1.493 | 0.132 | 0.230 |
| rostral anterior cingulate | 0.062 | -1.187 | 1.301 | 0.895 | 0.981 |
| rostral middle frontal | 0.408 | -0.263 | 1.113 | 0.230 | 0.333 |
| superior frontal | 0.299 | -0.517 | 1.178 | 0.474 | 0.663 |
| superior parietal | 0.576 | -0.312 | 1.446 | 0.195 | 0.455 |
| superior temporal | 0.909 | -0.093 | 1.942 | 0.073 | 0.127 |
| supramarginal | 0.815 | 0.103 | 1.638 | **0.027** | 0.062 ^ |
| frontal pole | 0.379 | -1.058 | 1.705 | 0.537 | 0.751 |
| temporal pole | 1.671 | -0.255 | 3.788 | 0.095 | 0.165 |
| transverse temporal | 1.560 | 0.194 | 2.986 | **0.026** | 0.061 ^ |
| insula | 1.105 | 0.206 | 2.016 | **0.020** | **0.047** |

**Supplemental Table 7. Effect modification of entorhinal FTP on the relationship between LC MRI signal intensity and cortical thickness for the entire sample, corrected for age, sex, and years of education.** Analyses were performed using bootstrapped linear regressions with cortical thickness regions as the dependent variable, LC MRI signal intensity interacted with EC FTP as the independent variables, and age, sex and years of education as covariates (n = 165). Reported regression coefficients are unstandardized, and P-values have been corrected for multiple comparisons using FDR. Significant P-values have been indicated in bold. P-values at trend level are indicated with ^. Significant P-values of regions in models with non-PVC data have been outlined by a blue box. Lower LC MRI signal intensity is associated with lower entorhinal, fusiform, inferior parietal, inferior temporal, lateral occipital, lingual, middle temporal, parahippocampal, precentral and insular cortical thickness, particularly in individuals with elevated EC FTP. Abbreviations: CI, confidence interval; FDR, False Discovery Rate; Bankssts, banks of superior temporal sulcus.

| **Cortical thickness region** | ***B*** | **Lower 95% CI** | **Upper 95% CI** | ***P*-value** | ***P_FDR_*-value** |
| --- | --- | --- | --- | --- | --- |
| bankssts | 0.785 | -0.407 | 1.941 | 0.193 | 0.450 |
| caudal anterior cingulate | -0.383 | -2.070 | 1.350 | 0.638 | 0.844 |
| caudal middle frontal | 1.047 | 0.023 | 2.116 | **0.046** | 0.106 |
| cuneus | 0.279 | -0.661 | 1.213 | 0.561 | 0.654 |
| entorhinal | 5.784 | 3.231 | 8.452 | **0.000** | **0.000** |
| fusiform | 1.979 | 1.016 | 2.949 | **0.000** | **0.000** |
| inferior parietal | 1.154 | 0.304 | 2.100 | **0.016** | **0.030** |
| inferior temporal | 1.099 | 0.135 | 2.087 | **0.016** | **0.037** |
| isthmus cingulate | 1.466 | -0.057 | 2.923 | 0.059 ^ | 0.102 |
| lateral occipital | 1.019 | 0.150 | 1.880 | **0.013** | **0.030** |
| lateral orbitofrontal | 0.696 | -0.190 | 1.567 | 0.121 | 0.211 |
| lingual | 1.095 | 0.296 | 1.870 | **0.005** | **0.012** |
| medial orbitofrontal | 0.708 | -0.374 | 1.758 | 0.201 | 0.449 |
| middle temporal | 1.679 | 0.798 | 2.650 | **0.002** | **0.005** |
| parahippocampal | 3.338 | 1.394 | 5.283 | **0.000** | **0.000** |
| paracentral | 1.019 | -0.161 | 2.274 | 0.097 | 0.226 |
| pars opercularis | 0.703 | -0.191 | 1.622 | 0.138 | 0.193 |
| pars orbitalis | 0.108 | -1.040 | 1.305 | 0.853 | 0.916 |
| pars triangularis | 1.064 | 0.116 | 2.020 | **0.026** | 0.061 ^ |
| pericalcarine | 0.781 | -0.165 | 1.637 | 0.105 | 0.245 |
| postcentral | 1.051 | -0.005 | 2.123 | 0.052 ^ | 0.120 |
| posterior cingulate | -0.512 | -1.773 | 0.763 | 0.383 | 0.421 |
| precentral | 1.230 | 0.118 | 2.518 | **0.030** | 0.053 ^ |
| precuneus | 0.669 | -0.216 | 1.760 | 0.166 | 0.289 |
| rostral anterior cingulate | -0.221 | -1.678 | 1.297 | 0.785 | 0.940 |
| rostral middle frontal | 0.489 | -0.285 | 1.290 | 0.220 | 0.314 |
| superior frontal | 0.473 | -0.525 | 1.497 | 0.335 | 0.468 |
| superior parietal | 0.717 | -0.308 | 1.800 | 0.186 | 0.393 |
| superior temporal | 0.931 | -0.175 | 2.134 | 0.103 | 0.180 |
| supramarginal | 0.995 | 0.090 | 1.920 | **0.036** | 0.083 |
| frontal pole | 0.417 | -1.270 | 1.989 | 0.617 | 0.835 |
| temporal pole | 2.577 | 0.220 | 5.131 | **0.026** | **0.046** |
| transverse temporal | 1.603 | -0.184 | 3.289 | 0.071 | 0.123 |
| insula | 1.377 | 0.272 | 2.530 | **0.016** | **0.037** |

**Supplemental Table 8. Effect modification of inferior temporal FTP on the relationship between LC MRI signal intensity and cortical thickness for the entire sample, corrected for age, sex, and years of education.** Analyses were performed using bootstrapped linear regressions with cortical thickness regions as the dependent variable, LC MRI signal intensity interacted with inferior temporal FTP as the independent variables, and age, sex and years of education as covariates (n = 165). Reported regression coefficients are unstandardized, and P-values have been corrected for multiple comparisons using FDR. Significant P-values have been indicated in bold, and P-values at trend level are indicated with ^. Significant P-values of regions in models with non-PVC data have been outlined by a blue box. Lower LC MRI signal intensity is associated with lower entorhinal, fusiform, inferior parietal, inferior temporal, lateral occipital, lingual, middle temporal, parahippocampal, temporal polar and insular cortical thickness, particularly in individuals with elevated inferior temporal FTP. Abbreviations: CI, confidence interval; FDR, False Discovery Rate; Bankssts, banks of superior temporal sulcus.

| **Cortical thickness region** | ***B*** | **Lower 95% CI** | **Upper 95% CI** | ***P*-value** | ***P_FDR_*-value** |
| --- | --- | --- | --- | --- | --- |
| bankssts | 0.640 | -0.290 | 1.668 | 0.199 | 0.397 |
| caudal anterior cingulate | -0.337 | -1.675 | 1.070 | 0.625 | 0.727 |
| caudal middle frontal | 0.517 | -0.325 | 1.375 | 0.238 | 0.377 |
| cuneus | 0.172 | -0.606 | 0.928 | 0.626 | 0.644 |
| entorhinal | 4.334 | 2.447 | 6.303 | **0.000** | **0.000** |
| fusiform | 1.392 | 0.571 | 2.183 | **0.000** | **0.000** |
| inferior parietal | 1.001 | 0.305 | 1.799 | **0.003** | **0.005** |
| inferior temporal | 1.042 | 0.231 | 1.884 | **0.012** | **0.032** |
| isthmus cingulate | 1.082 | -0.132 | 2.271 | 0.087 | 0.133 |
| lateral occipital | 1.068 | 0.393 | 1.768 | **0.003** | **0.008** |
| lateral orbitofrontal | 0.581 | -0.121 | 1.296 | 0.110 | 0.293 |
| lingual | 0.869 | 0.210 | 1.532 | **0.012** | **0.035** |
| medial orbitofrontal | 0.555 | -0.246 | 1.371 | 0.200 | 0.400 |
| middle temporal | 1.227 | 0.439 | 2.063 | **0.002** | **0.004** |
| parahippocampal | 1.949 | 0.329 | 3.675 | **0.017** | **0.044** |
| paracentral | 0.762 | -0.186 | 1.812 | 0.113 | 0.300 |
| pars opercularis | 0.507 | -0.286 | 1.356 | 0.177 | 0.251 |
| pars orbitalis | -0.270 | -1.258 | 0.722 | 0.623 | 0.851 |
| pars triangularis | 0.552 | -0.257 | 1.400 | 0.170 | 0.387 |
| pericalcarine | 0.689 | -0.070 | 1.397 | 0.072 | 0.193 |
| postcentral | 0.558 | -0.320 | 1.393 | 0.208 | 0.415 |
| posterior cingulate | -0.181 | -1.165 | 0.820 | 0.701 | 0.753 |
| precentral | 1.071 | 0.126 | 2.124 | **0.028** | 0.056 ^ |
| precuneus | 0.528 | -0.235 | 1.402 | 0.193 | 0.305 |
| rostral anterior cingulate | -0.072 | -1.203 | 1.206 | 0.945 | 0.945 |
| rostral middle frontal | 0.357 | -0.290 | 1.032 | 0.284 | 0.378 |
| superior frontal | 0.228 | -0.548 | 1.053 | 0.569 | 0.758 |
| superior parietal | 0.497 | -0.353 | 1.386 | 0.249 | 0.470 |
| superior temporal | 0.860 | -0.128 | 1.889 | 0.090 | 0.179 |
| supramarginal | 0.744 | -0.010 | 1.580 | 0.053 ^ | 0.106 |
| frontal pole | 0.303 | -1.244 | 1.647 | 0.650 | 0.831 |
| temporal pole | 1.707 | -0.251 | 3.836 | 0.087 | 0.174 |
| transverse temporal | 1.522 | 0.125 | 2.948 | **0.028** | 0.073 |
| insula | 1.069 | 0.165 | 1.995 | **0.018** | **0.048** |

**Supplemental Table 9. Effect modification of entorhinal FTP on the relationship between LC MRI signal intensity and cortical thickness for the entire sample, corrected for neocortical PiB, age, sex, and years of education.** Analyses were performed using bootstrapped linear regressions with cortical thickness regions as the dependent variable, LC MRI signal intensity interacted with EC FTP as the independent variables, and neocortical PiB, age, sex and years of education as covariates (n = 165). Reported regression coefficients are unstandardized, and P-values have been corrected for multiple comparisons using FDR. Significant P-values have been indicated in bold, and P-values at trend level are indicated with ^. Significant P-values of regions in models with non-PVC data have been outlined by a blue box. Lower LC MRI signal intensity is associated with lower entorhinal, fusiform, inferior parietal, inferior temporal, lateral occipital, lingual, middle temporal, parahippocampal and insular cortical thickness, particularly in individuals with elevated EC FTP. Abbreviations: CI, confidence interval; FDR, False Discovery Rate; Bankssts, banks of superior temporal sulcus.

| **Cortical thickness region** | ***B*** | **Lower 95% CI** | **Upper 95% CI** | ***P*-value** | ***P_FDR_*-value** |
| --- | --- | --- | --- | --- | --- |
| bankssts | 0.666 | -0.473 | 1.912 | 0.271 | 0.541 |
| caudal anterior cingulate | -0.640 | -2.293 | 1.257 | 0.520 | 0.628 |
| caudal middle frontal | 0.963 | -0.061 | 2.028 | 0.059 ^ | 0.157 |
| cuneus | 0.237 | -0.700 | 1.147 | 0.588 | 0.630 |
| entorhinal | 5.950 | 3.316 | 8.695 | **0.000** | **0.000** |
| fusiform | 1.833 | 0.921 | 2.778 | **0.000** | **0.000** |
| inferior parietal | 1.032 | 0.183 | 2.042 | **0.015** | **0.028** |
| inferior temporal | 1.094 | 0.153 | 2.195 | **0.023** | 0.061 ^ |
| isthmus cingulate | 1.306 | -0.183 | 2.801 | 0.074 | 0.118 |
| lateral occipital | 0.922 | 0.072 | 1.792 | **0.039** | 0.076 |
| lateral orbitofrontal | 0.678 | -0.254 | 1.557 | 0.143 | 0.286 |
| lingual | 1.030 | 0.206 | 1.823 | **0.014** | **0.037** |
| medial orbitofrontal | 0.599 | -0.451 | 1.636 | 0.259 | 0.485 |
| middle temporal | 1.625 | 0.720 | 2.622 | **0.000** | **0.000** |
| parahippocampal | 3.191 | 1.186 | 5.155 | **0.003** | **0.008** |
| paracentral | 0.967 | -0.172 | 2.234 | 0.104 | 0.277 |
| pars opercularis | 0.673 | -0.234 | 1.579 | 0.165 | 0.264 |
| pars orbitalis | 0.082 | -1.129 | 1.329 | 0.912 | 0.984 |
| pars triangularis | 1.016 | 0.052 | 2.018 | **0.037** | 0.099 |
| pericalcarine | 0.689 | -0.299 | 1.548 | 0.159 | 0.332 |
| postcentral | 1.002 | -0.041 | 2.099 | 0.063 ^ | 0.167 |
| posterior cingulate | -0.672 | -1.849 | 0.513 | 0.260 | 0.271 |
| precentral | 1.169 | 0.001 | 2.460 | 0.050 ^ | 0.100 |
| precuneus | 0.521 | -0.425 | 1.616 | 0.302 | 0.414 |
| rostral anterior cingulate | -0.484 | -1.969 | 1.081 | 0.556 | 0.644 |
| rostral middle frontal | 0.402 | -0.420 | 1.240 | 0.322 | 0.413 |
| superior frontal | 0.366 | -0.568 | 1.409 | 0.487 | 0.634 |
| superior parietal | 0.582 | -0.530 | 1.697 | 0.295 | 0.483 |
| superior temporal | 0.903 | -0.345 | 2.119 | 0.146 | 0.292 |
| supramarginal | 0.886 | -0.008 | 1.849 | 0.053 ^ | 0.105 |
| frontal pole | 0.276 | -1.536 | 1.961 | 0.727 | 0.861 |
| temporal pole | 2.707 | 0.301 | 5.363 | **0.024** | **0.048** |
| transverse temporal | 1.540 | -0.085 | 3.279 | 0.074 | 0.148 |
| insula | 1.339 | 0.194 | 2.424 | **0.023** | 0.060 ^ |

**Supplemental Table 10. Effect modification of inferior temporal FTP on the relationship between LC MRI signal intensity and cortical thickness for the entire sample, corrected for neocortical PiB, age, sex, and years of education.** Analyses were performed using bootstrapped linear regressions with cortical thickness regions as the dependent variable, LC MRI signal intensity interacted with inferior temporal FTP as the independent variables, and neocortical PiB, age, sex and years of education as covariates (n = 165). Reported regression coefficients are unstandardized, and P-values have been corrected for multiple comparisons using FDR. Significant P-values have been indicated in bold, and P-values at trend level are indicated with ^. Significant P-values of regions in models with non-PVC data have been outlined by a blue box. Lower LC MRI signal intensity is associated with lower entorhinal, fusiform, inferior parietal, lingual, middle temporal, parahippocampal and temporal polar cortical thickness, particularly in individuals with elevated inferior temporal FTP. Abbreviations: CI, confidence interval; FDR, False Discovery Rate; Bankssts, banks of superior temporal sulcus.

| **Cortical thickness region** | ***B*** | **Lower 95% CI** | **Upper 95% CI** | ***P*-value** | ***P_FDR_*-value** |
| --- | --- | --- | --- | --- | --- |
| bankssts | 0.410 | -0.879 | 1.802 | 0.548 | 0.767 |
| caudal anterior cingulate | -0.883 | -2.752 | 1.199 | 0.403 | 0.627 |
| caudal middle frontal | -0.036 | -1.196 | 1.194 | 0.947 | 0.988 |
| cuneus | 0.172 | -0.694 | 1.161 | 0.734 | 0.814 |
| entorhinal | 6.601 | 4.014 | 9.089 | **0.000** | **0.000** |
| fusiform | 1.450 | 0.407 | 2.563 | **0.008** | **0.018** |
| inferior parietal | 0.889 | -0.078 | 1.854 | 0.077 | 0.107 |
| inferior temporal | 0.712 | -0.300 | 1.654 | 0.167 | 0.292 |
| isthmus cingulate | 1.702 | 0.224 | 3.242 | **0.023** | **0.040** |
| lateral occipital | 0.909 | 0.025 | 1.861 | 0.045 | 0.083 |
| lateral orbitofrontal | 0.746 | -0.171 | 1.726 | 0.122 | 0.265 |
| lingual | 0.354 | -0.517 | 1.207 | 0.417 | 0.592 |
| medial orbitofrontal | 1.147 | -0.023 | 2.252 | 0.057 ^ | 0.133 |
| middle temporal | 1.093 | 0.136 | 2.086 | **0.025** | 0.058 ^ |
| parahippocampal | 1.027 | -1.117 | 3.357 | 0.370 | 0.517 |
| paracentral | -0.367 | -1.581 | 0.961 | 0.571 | 0.665 |
| pars opercularis | 0.460 | -0.537 | 1.492 | 0.352 | 0.493 |
| pars orbitalis | 0.257 | -1.111 | 1.600 | 0.708 | 0.797 |
| pars triangularis | 0.678 | -0.369 | 1.727 | 0.192 | 0.336 |
| pericalcarine | 0.368 | -0.526 | 1.262 | 0.427 | 0.593 |
| postcentral | 0.489 | -0.704 | 1.712 | 0.399 | 0.697 |
| posterior cingulate | -0.362 | -1.570 | 0.911 | 0.561 | 0.603 |
| precentral | 0.114 | -1.081 | 1.453 | 0.854 | 0.920 |
| precuneus | -0.114 | -1.200 | 1.058 | 0.825 | 0.923 |
| rostral anterior cingulate | -0.505 | -2.185 | 1.123 | 0.521 | 0.544 |
| rostral middle frontal | 0.044 | -0.850 | 1.002 | 0.925 | 0.973 |
| superior frontal | -0.016 | -1.072 | 1.153 | 0.943 | 0.986 |
| superior parietal | 0.092 | -1.099 | 1.317 | 0.896 | 0.964 |
| superior temporal | 1.017 | -0.238 | 2.527 | 0.118 | 0.206 |
| supramarginal | 0.758 | -0.158 | 1.873 | 0.112 | 0.159 |
| frontal pole | 1.324 | -0.554 | 3.062 | 0.154 | 0.270 |
| temporal pole | 4.104 | 1.693 | 6.542 | **0.000** | **0.000** |
| transverse temporal | 0.592 | -1.186 | 2.656 | 0.555 | 0.776 |
| insula | 0.720 | -0.437 | 1.946 | 0.226 | 0.396 |

**Supplemental Table 11.** **Effect modification of entorhinal FTP on the relationship between LC MRI signal intensity and cortical thickness for the CDR = 0 sample, corrected for age, sex, and years of education.** Analyses were performed using bootstrapped linear regressions with cortical thickness regions as the dependent variable, LC MRI signal intensity interacted with EC FTP as the independent variables, and age, sex and years of education as covariates (n = 149). Reported regression coefficients are unstandardized, and P-values have been corrected for multiple comparisons using FDR. Significant P-values have been indicated in bold, and P-values at trend level are indicated with ^. Significant P-values of regions in models with non-PVC data have been outlined by a blue box. In individuals with CDR = 0, lower LC MRI signal intensity is associated with lower entorhinal, fusiform, isthmus cingulate and temporal polar cortical thickness, particularly in individuals with elevated EC FTP. Abbreviations: CI, confidence interval; FDR, False Discovery Rate; Bankssts, banks of superior temporal sulcus.

| **Cortical thickness region** | ***B*** | **Lower 95% CI** | **Upper 95% CI** | ***P*-value** | ***P_FDR_*-value** |
| --- | --- | --- | --- | --- | --- |
| bankssts | 0.530 | -0.673 | 1.773 | 0.387 | 0.558 |
| caudal anterior cingulate | 0.244 | -1.545 | 1.958 | 0.786 | 0.786 |
| caudal middle frontal | 1.038 | 0.040 | 2.083 | **0.041** | 0.096 |
| cuneus | 0.715 | -0.237 | 1.637 | 0.147 | 0.256 |
| entorhinal | 5.199 | 2.514 | 8.078 | **0.000** | **0.000** |
| fusiform | 1.829 | 0.822 | 2.919 | **0.000** | **0.000** |
| inferior parietal | 0.979 | 0.069 | 1.932 | **0.030** | 0.067 ^ |
| inferior temporal | 1.787 | 0.768 | 2.797 | **0.000** | **0.000** |
| isthmus cingulate | 0.359 | -1.097 | 1.823 | 0.589 | 0.687 |
| lateral occipital | 1.485 | 0.641 | 2.338 | **0.000** | **0.000** |
| lateral orbitofrontal | 1.067 | 0.178 | 1.907 | **0.022** | 0.050 ^ |
| lingual | 1.072 | 0.249 | 1.881 | **0.011** | **0.026** |
| medial orbitofrontal | 0.543 | -0.476 | 1.536 | 0.305 | 0.599 |
| middle temporal | 1.629 | 0.600 | 2.666 | **0.003** | **0.006** |
| parahippocampal | 2.789 | 0.730 | 4.875 | **0.011** | **0.025** |
| paracentral | 2.020 | 0.902 | 3.249 | **0.002** | **0.005** |
| pars opercularis | 0.770 | -0.214 | 1.666 | 0.122 | 0.171 |
| pars orbitalis | 0.689 | -0.531 | 2.000 | 0.271 | 0.473 |
| pars triangularis | 0.754 | -0.313 | 1.757 | 0.150 | 0.349 |
| pericalcarine | 1.212 | 0.258 | 2.083 | **0.012** | **0.028** |
| postcentral | 0.599 | -0.521 | 1.741 | 0.281 | 0.610 |
| posterior cingulate | 0.080 | -1.083 | 1.282 | 0.910 | 0.972 |
| precentral | 1.645 | 0.478 | 2.831 | **0.006** | **0.011** |
| precuneus | 0.688 | -0.256 | 1.716 | 0.177 | 0.293 |
| rostral anterior cingulate | 1.027 | -0.454 | 2.508 | 0.160 | 0.302 |
| rostral middle frontal | 0.915 | 0.070 | 1.745 | **0.030** | 0.070 |
| superior frontal | 0.559 | -0.410 | 1.588 | 0.279 | 0.390 |
| superior parietal | 0.428 | -0.664 | 1.515 | 0.417 | 0.570 |
| superior temporal | 1.605 | 0.305 | 2.901 | **0.016** | **0.028** |
| supramarginal | 0.835 | -0.146 | 1.813 | 0.103 | 0.180 |
| frontal pole | -0.496 | -2.282 | 1.199 | 0.605 | 0.769 |
| temporal pole | 2.147 | -0.448 | 4.845 | 0.102 | 0.179 |
| transverse temporal | 1.985 | 0.231 | 3.781 | **0.022** | 0.051 ^ |
| insula | 2.126 | 1.009 | 3.281 | **0.000** | **0.000** |

**Supplemental Table 12. Effect modification of neocortical PiB on the relationship between LC MRI signal intensity and cortical thickness for the entire sample, corrected for age, sex, and years of education.** Analyses were performed using bootstrapped linear regressions with cortical thickness regions as the dependent variable, LC MRI signal intensity interacted with neocortical PiB as the independent variables, and age, sex and years of education as covariates (n = 165). Reported regression coefficients are unstandardized, and P-values have been corrected for multiple comparisons using FDR. Significant P-values have been indicated in bold, and P-values at trend level are indicated with ^. Significant P-values of regions in models with non-PVC data have been outlined by a blue box. Lower LC MRI signal intensity is associated with lower entorhinal, fusiform, inferior temporal, lateral occipital, lingual, middle temporal, parahippocampal, paracentral, pericalcarine, precentral, superior temporal and insular cortical thickness, particularly in individuals with elevated neocortical PiB. Abbreviations: CI, confidence interval; FDR, False Discovery Rate; Bankssts, banks of superior temporal sulcus.

| **Cortical thickness region** | ***B*** | **Lower 95% CI** | **Upper 95% CI** | ***P*-value** | ***P_FDR_*-value** |
| --- | --- | --- | --- | --- | --- |
| bankssts | 0.507 | -0.778 | 1.794 | 0.438 | 0.649 |
| caudal anterior cingulate | 0.170 | -1.647 | 1.946 | 0.871 | 0.871 |
| caudal middle frontal | 0.933 | -0.193 | 2.045 | 0.096 | 0.239 |
| cuneus | 0.767 | -0.171 | 1.722 | 0.105 | 0.210 |
| entorhinal | 3.609 | 0.975 | 6.102 | **0.005** | **0.007** |
| fusiform | 1.614 | 0.581 | 2.697 | **0.003** | **0.007** |
| inferior parietal | 0.942 | -0.005 | 1.890 | 0.052 ^ | 0.104 |
| inferior temporal | 1.573 | 0.537 | 2.652 | **0.002** | **0.005** |
| isthmus cingulate | 0.189 | -1.346 | 1.766 | 0.758 | 0.866 |
| lateral occipital | 1.390 | 0.540 | 2.279 | **0.002** | **0.005** |
| lateral orbitofrontal | 0.852 | -0.082 | 1.759 | 0.076 | 0.151 |
| lingual | 1.102 | 0.307 | 1.922 | **0.012** | **0.032** |
| medial orbitofrontal | 0.576 | -0.526 | 1.672 | 0.312 | 0.729 |
| middle temporal | 1.460 | 0.482 | 2.498 | **0.003** | **0.008** |
| parahippocampal | 2.479 | 0.333 | 4.656 | **0.026** | 0.051 ^ |
| paracentral | 1.897 | 0.713 | 3.197 | **0.000** | **0.000** |
| pars opercularis | 0.705 | -0.324 | 1.665 | 0.162 | 0.258 |
| pars orbitalis | 0.528 | -0.715 | 1.748 | 0.407 | 0.589 |
| pars triangularis | 0.674 | -0.290 | 1.697 | 0.181 | 0.362 |
| pericalcarine | 1.268 | 0.309 | 2.166 | **0.012** | **0.031** |
| postcentral | 0.625 | -0.506 | 1.779 | 0.260 | 0.693 |
| posterior cingulate | -0.052 | -1.347 | 1.199 | 0.930 | 0.993 |
| precentral | 1.620 | 0.343 | 2.877 | **0.007** | **0.013** |
| precuneus | 0.695 | -0.302 | 1.814 | 0.188 | 0.344 |
| rostral anterior cingulate | 1.136 | -0.404 | 2.671 | 0.139 | 0.359 |
| rostral middle frontal | 0.826 | 0.020 | 1.658 | **0.044** | 0.116 |
| superior frontal | 0.478 | -0.526 | 1.510 | 0.382 | 0.526 |
| superior parietal | 0.407 | -0.743 | 1.525 | 0.486 | 0.750 |
| superior temporal | 1.461 | 0.200 | 2.733 | **0.022** | **0.044** |
| supramarginal | 0.712 | -0.276 | 1.716 | 0.181 | 0.294 |
| frontal pole | -0.594 | -2.366 | 1.174 | 0.519 | 0.677 |
| temporal pole | 1.493 | -1.025 | 4.151 | 0.250 | 0.407 |
| transverse temporal | 1.996 | 0.204 | 3.717 | **0.034** | 0.089 |
| insula | 2.092 | 0.924 | 3.254 | **0.002** | **0.005** |

**Supplemental Table 13. Effect modification of neocortical PiB on the relationship between LC MRI signal intensity and cortical thickness for the entire sample, corrected for entorhinal FTP, age, sex, and years of education.** Analyses were performed using bootstrapped linear regressions with cortical thickness regions as the dependent variable, LC MRI signal intensity interacted with neocortical PiB as the independent variables, and entorhinal FTP, age, sex and years of education as covariates (n = 165). Reported regression coefficients are unstandardized, and P-values have been corrected for multiple comparisons using FDR. Significant P-values have been indicated in bold, and P-values at trend level are indicated with ^. Significant P-values of regions in models with non-PVC data have been outlined by a blue box. Lower LC MRI signal intensity is associated with lower entorhinal, fusiform, inferior temporal, lateral occipital, lingual, middle temporal, paracentral, pericalcarine, precentral, superior temporal and insular cortical thickness, particularly in individuals with elevated neocortical PiB. Abbreviations: CI, confidence interval; FDR, False Discovery Rate; Bankssts, banks of superior temporal sulcus.

| **Cortical thickness region** | ***B*** | **Lower 95% CI** | **Upper 95% CI** | ***P*-value** | ***P_FDR_*-value** |
| --- | --- | --- | --- | --- | --- |
| bankssts | 0.187 | -1.205 | 1.607 | 0.831 | 0.831 |
| caudal anterior cingulate | 0.323 | -1.707 | 2.307 | 0.768 | 0.768 |
| caudal middle frontal | 1.059 | -0.094 | 2.245 | 0.078 | 0.181 |
| cuneus | 1.042 | 0.088 | 2.071 | **0.036** | 0.084 |
| entorhinal | 4.855 | 2.028 | 7.931 | **0.002** | **0.003** |
| fusiform | 1.808 | 0.708 | 2.974 | **0.002** | **0.005** |
| inferior parietal | 1.013 | -0.054 | 2.036 | 0.064 ^ | 0.148 |
| inferior temporal | 1.490 | 0.325 | 2.591 | **0.014** | **0.032** |
| isthmus cingulate | 1.246 | -0.344 | 2.848 | 0.129 | 0.181 |
| lateral occipital | 1.109 | 0.160 | 2.125 | **0.024** | 0.056 ^ |
| lateral orbitofrontal | 1.087 | 0.069 | 2.059 | **0.034** | 0.079 |
| lingual | 0.739 | -0.160 | 1.674 | 0.114 | 0.199 |
| medial orbitofrontal | 0.222 | -0.926 | 1.337 | 0.681 | 0.960 |
| middle temporal | 1.666 | 0.588 | 2.802 | **0.002** | **0.005** |
| parahippocampal | 2.137 | -0.326 | 4.442 | 0.070 | 0.123 |
| paracentral | 2.270 | 0.961 | 3.580 | **0.000** | **0.000** |
| pars opercularis | 1.065 | 0.025 | 2.100 | **0.042** | 0.088 |
| pars orbitalis | 0.635 | -0.690 | 2.021 | 0.369 | 0.645 |
| pars triangularis | 1.226 | 0.164 | 2.318 | **0.026** | 0.060 ^ |
| pericalcarine | 0.885 | -0.123 | 1.892 | 0.080 | 0.139 |
| postcentral | 0.852 | -0.367 | 2.058 | 0.162 | 0.377 |
| posterior cingulate | 0.063 | -1.303 | 1.439 | 0.942 | 0.942 |
| precentral | 1.671 | 0.313 | 3.089 | **0.013** | **0.022** |
| precuneus | 0.873 | -0.193 | 2.019 | 0.118 | 0.207 |
| rostral anterior cingulate | 1.697 | 0.093 | 3.364 | **0.040** | 0.093 |
| rostral middle frontal | 0.758 | -0.125 | 1.681 | 0.109 | 0.191 |
| superior frontal | 0.834 | -0.255 | 1.894 | 0.131 | 0.229 |
| superior parietal | 0.608 | -0.555 | 1.734 | 0.306 | 0.714 |
| superior temporal | 2.140 | 0.826 | 3.539 | **0.003** | **0.005** |
| supramarginal | 1.332 | 0.230 | 2.387 | **0.018** | **0.041** |
| frontal pole | -0.102 | -1.971 | 1.805 | 0.933 | 0.984 |
| temporal pole | 2.932 | 0.402 | 5.834 | **0.027** | **0.046** |
| transverse temporal | 2.704 | 0.856 | 4.687 | **0.006** | **0.014** |
| insula | 2.297 | 1.032 | 3.546 | **0.002** | **0.004** |

**Supplemental Table 14. Effect modification of neocortical PiB status on the relationship between LC MRI signal intensity and cortical thickness for the entire sample, corrected for age, sex, and years of education.** Analyses were performed using bootstrapped linear regressions with cortical thickness regions as the dependent variable, LC MRI signal intensity interacted with dichotomous neocortical PiB status as the independent variables, and age, sex and years of education as covariates (n = 165). Reported regression coefficients are unstandardized, and P-values have been corrected for multiple comparisons using FDR. Significant P-values have been indicated in bold. P-values at trend level are indicated with ^. Significant P-values of regions in models with non-PVC data have been outlined by a blue box. Lower LC MRI signal intensity is associated with lower entorhinal, fusiform, inferior temporal, middle temporal, paracentral, precentral, superior temporal, supramarginal, temporal polar, transverse temporal and insular cortical thickness, particularly in individuals with elevated neocortical PiB. Abbreviations: CI, confidence interval; FDR, False Discovery Rate; Bankssts, banks of superior temporal sulcus.

| **Cortical thickness region** | ***B*** | **Lower 95% CI** | **Upper 95% CI** | ***P*-value** | ***P_FDR_*-value** |
| --- | --- | --- | --- | --- | --- |
| bankssts | -0.142 | -1.982 | 1.803 | 0.869 | 0.917 |
| caudal anterior cingulate | 0.022 | -2.917 | 2.994 | 0.978 | 0.978 |
| caudal middle frontal | -0.416 | -2.148 | 1.230 | 0.655 | 0.766 |
| cuneus | 0.179 | -1.224 | 1.587 | 0.799 | 0.799 |
| entorhinal | 3.606 | -0.606 | 7.815 | 0.093 | 0.130 |
| fusiform | 1.216 | -0.256 | 2.815 | 0.103 | 0.144 |
| inferior parietal | 0.719 | -0.708 | 2.179 | 0.308 | 0.431 |
| inferior temporal | 0.645 | -0.798 | 2.178 | 0.395 | 0.683 |
| isthmus cingulate | -0.541 | -2.868 | 1.804 | 0.654 | 0.787 |
| lateral occipital | 0.756 | -0.598 | 2.091 | 0.269 | 0.321 |
| lateral orbitofrontal | 0.773 | -0.660 | 2.257 | 0.284 | 0.496 |
| lingual | -0.180 | -1.379 | 1.080 | 0.778 | 0.785 |
| medial orbitofrontal | 0.544 | -1.216 | 2.319 | 0.542 | 0.963 |
| middle temporal | 0.587 | -0.870 | 2.083 | 0.423 | 0.561 |
| parahippocampal | 0.927 | -2.457 | 4.416 | 0.577 | 0.673 |
| paracentral | 0.783 | -1.070 | 2.639 | 0.403 | 0.687 |
| pars opercularis | 1.000 | -0.438 | 2.483 | 0.168 | 0.298 |
| pars orbitalis | 1.829 | -0.205 | 3.798 | 0.078 | 0.136 |
| pars triangularis | 0.371 | -1.206 | 1.911 | 0.663 | 0.772 |
| pericalcarine | 0.986 | -0.421 | 2.370 | 0.183 | 0.296 |
| postcentral | 0.000 | -1.824 | 1.714 | 0.988 | 0.996 |
| posterior cingulate | -0.681 | -2.402 | 1.177 | 0.477 | 0.507 |
| precentral | 0.030 | -1.943 | 1.892 | 0.967 | 0.967 |
| precuneus | 0.003 | -1.621 | 1.654 | 0.996 | 0.996 |
| rostral anterior cingulate | 1.803 | -0.625 | 4.232 | 0.142 | 0.250 |
| rostral middle frontal | 0.326 | -1.002 | 1.676 | 0.654 | 0.738 |
| superior frontal | -0.043 | -1.681 | 1.587 | 0.953 | 0.982 |
| superior parietal | -0.452 | -2.163 | 1.288 | 0.591 | 0.957 |
| superior temporal | 1.840 | -0.101 | 3.909 | 0.063 ^ | 0.109 |
| supramarginal | 0.683 | -0.767 | 2.193 | 0.342 | 0.454 |
| frontal pole | 0.475 | -2.357 | 3.306 | 0.716 | 0.900 |
| temporal pole | 3.287 | -0.422 | 7.188 | 0.096 | 0.168 |
| transverse temporal | 1.439 | -1.513 | 4.133 | 0.320 | 0.560 |
| insula | 1.876 | 0.100 | 3.693 | **0.036** | 0.084 |

**Supplemental Table 15.** **Effect modification of neocortical PiB on the relationship between LC MRI signal intensity and cortical thickness for the CDR = 0 sample, corrected for age, sex, and years of education.** Analyses were performed using bootstrapped linear regressions with cortical thickness regions as the dependent variable, LC MRI signal intensity interacted with neocortical PiB as the independent variables, and age, sex and years of education as covariates (n = 149). Reported regression coefficients are unstandardized, and P-values have been corrected for multiple comparisons using FDR. Significant P-values have been indicated in bold, and P-values at trend level are indicated with ^. Significant P-values of regions in models with non-PVC data have been outlined by a blue box. In individuals with CDR = 0, LC MRI signal intensity is not associated with cortical thickness, regardless of neocortical PiB level. Abbreviations: CI, confidence interval; FDR, False Discovery Rate; Bankssts, banks of superior temporal sulcus.


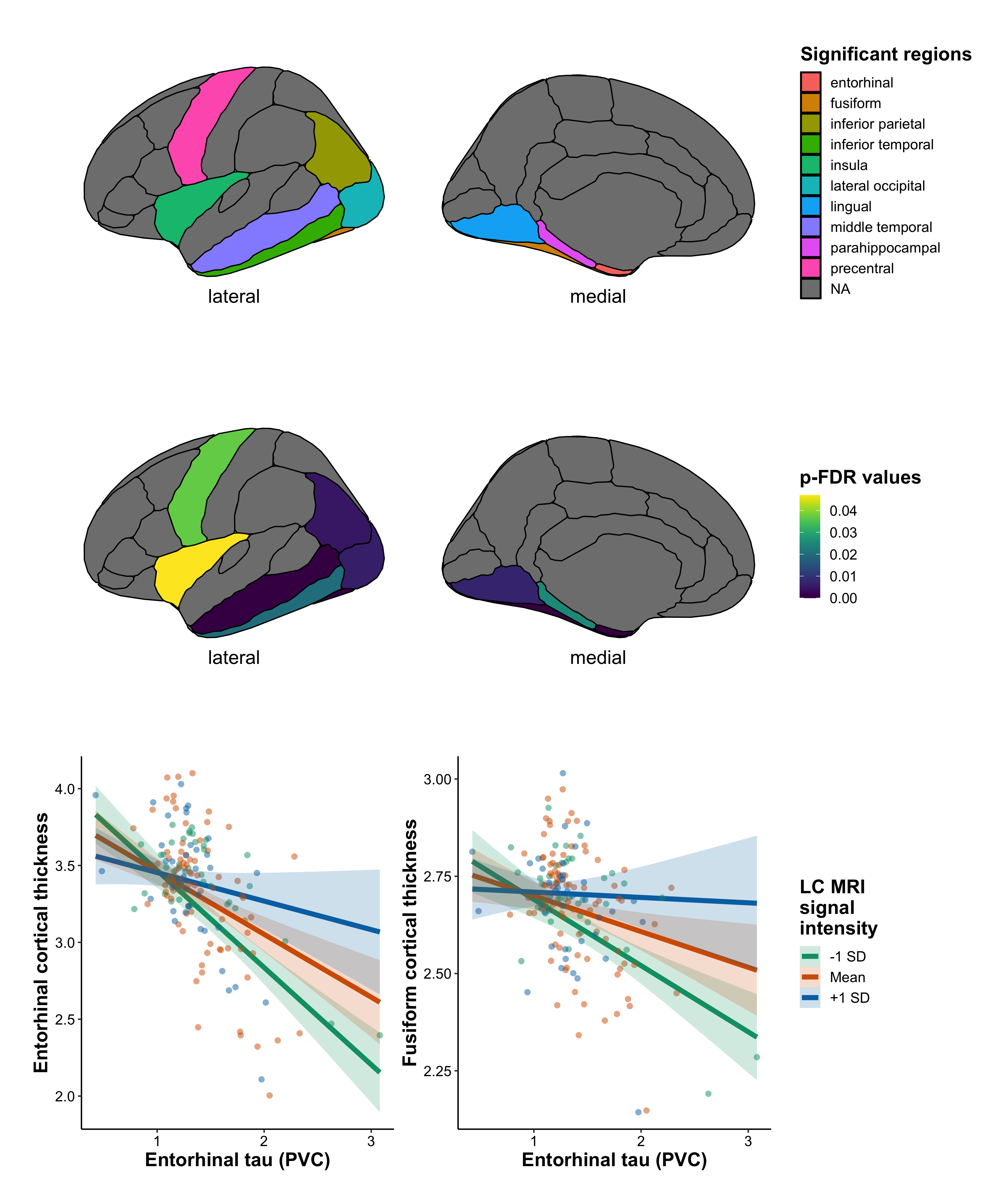
**Lower LC MRI signal intensity is related to lower entorhinal cortical thickness in individuals with low beta-amyloid**

| Cortical thickness region | *B* | Lower 95% CI | Upper 95% CI | *P*-value | *P_FDR_*-value |
| --- | --- | --- | --- | --- | --- |
| bankssts | 0.562 | -0.890 | 2.180 | 0.445 | 0.675 |
| caudal anterior cingulate | -0.385 | -2.510 | 1.714 | 0.738 | 0.890 |
| caudal middle frontal | 0.000 | -1.130 | 1.211 | 0.977 | 0.977 |
| cuneus | -0.482 | -1.632 | 0.677 | 0.399 | 0.567 |
| entorhinal | 5.419 | 2.516 | 8.160 | **0.003** | **0.004** |
| fusiform | 1.052 | 0.007 | 2.108 | **0.048** | 0.083 |
| inferior parietal | 0.787 | -0.222 | 1.958 | 0.140 | 0.196 |
| inferior temporal | 0.481 | -0.619 | 1.669 | 0.403 | 0.646 |
| isthmus cingulate | 1.224 | -0.608 | 3.004 | 0.174 | 0.243 |
| lateral occipital | 0.937 | -0.017 | 1.915 | 0.058 ^ | 0.101 |
| lateral orbitofrontal | 0.643 | -0.383 | 1.747 | 0.210 | 0.326 |
| lingual | 0.264 | -0.764 | 1.249 | 0.603 | 0.855 |
| medial orbitofrontal | 0.815 | -0.503 | 1.997 | 0.213 | 0.465 |
| middle temporal | 0.767 | -0.274 | 1.922 | 0.146 | 0.255 |
| parahippocampal | 1.201 | -1.025 | 3.486 | 0.284 | 0.395 |
| paracentral | -0.548 | -1.756 | 0.823 | 0.406 | 0.561 |
| pars opercularis | 0.067 | -0.961 | 1.226 | 0.902 | 0.971 |
| pars orbitalis | 0.026 | -1.277 | 1.408 | 0.956 | 0.970 |
| pars triangularis | 0.496 | -0.682 | 1.600 | 0.375 | 0.524 |
| pericalcarine | 0.056 | -1.100 | 1.111 | 0.902 | 0.902 |
| postcentral | 0.471 | -0.858 | 1.782 | 0.471 | 0.811 |
| posterior cingulate | -0.476 | -1.954 | 1.087 | 0.527 | 0.559 |
| precentral | -0.114 | -1.516 | 1.349 | 0.855 | 0.855 |
| precuneus | -0.126 | -1.165 | 1.038 | 0.800 | 0.980 |
| rostral anterior cingulate | -0.870 | -2.863 | 0.999 | 0.382 | 0.619 |
| rostral middle frontal | -0.079 | -1.000 | 0.907 | 0.851 | 0.851 |
| superior frontal | -0.068 | -1.113 | 1.074 | 0.862 | 0.893 |
| superior parietal | 0.282 | -0.903 | 1.477 | 0.639 | 0.886 |
| superior temporal | 0.246 | -1.137 | 1.758 | 0.792 | 0.924 |
| supramarginal | 0.451 | -0.677 | 1.688 | 0.441 | 0.441 |
| frontal pole | 1.585 | -0.507 | 3.471 | 0.110 | 0.192 |
| temporal pole | 2.577 | 0.017 | 5.593 | **0.045** | 0.079 |
| transverse temporal | 0.829 | -1.237 | 2.880 | 0.456 | 0.671 |
| insula | -0.144 | -1.426 | 1.197 | 0.793 | 0.793 |

**Supplemental Table 16. Effect modification of entorhinal FTP on the relationship between LC MRI signal intensity and cortical thickness for the low beta-amyloid sample, corrected for age, sex, and years of education.** Analyses were performed using bootstrapped linear regressions with cortical thickness regions as the dependent variable, LC MRI signal intensity interacted with EC FTP as the independent variables, and age, sex and years of education as covariates (n = 112). Reported regression coefficients are unstandardized, and P-values have been corrected for multiple comparisons using FDR. Significant P-values have been indicated in bold, and P-values at trend level are indicated with ^. Significant P-values of regions in models with non-PVC data have been outlined by a blue box. In individuals with low beta-amyloid, lower LC MRI signal intensity is associated with lower entorhinal cortical thickness, particularly in individuals with elevated EC FTP. Abbreviations: CI, confidence interval; FDR, False Discovery Rate; Bankssts, banks of superior temporal sulcus.

| Cortical thickness region | *B* | Lower 95% CI | Upper 95% CI | *P*-value | *P_FDR_*-value |
| --- | --- | --- | --- | --- | --- |
| bankssts | -0.065 | -1.888 | 1.861 | 0.951 | 0.951 |
| caudal anterior cingulate | -0.030 | -2.721 | 2.890 | 0.979 | 0.979 |
| caudal middle frontal | 0.662 | -0.828 | 2.248 | 0.402 | 0.562 |
| cuneus | -0.540 | -2.003 | 0.964 | 0.462 | 0.686 |
| entorhinal | 5.767 | 1.880 | 9.861 | **0.000** | **0.000** |
| fusiform | 1.049 | -0.292 | 2.359 | 0.112 | 0.196 |
| inferior parietal | 0.912 | -0.442 | 2.331 | 0.205 | 0.287 |
| inferior temporal | 0.823 | -0.603 | 2.286 | 0.225 | 0.525 |
| isthmus cingulate | 1.645 | -0.658 | 3.873 | 0.142 | 0.213 |
| lateral occipital | 0.942 | -0.350 | 2.265 | 0.149 | 0.261 |
| lateral orbitofrontal | 0.320 | -0.956 | 1.765 | 0.634 | 0.794 |
| lingual | 0.182 | -1.062 | 1.378 | 0.741 | 0.992 |
| medial orbitofrontal | 0.644 | -1.014 | 2.182 | 0.417 | 0.751 |
| middle temporal | 1.249 | -0.149 | 2.620 | 0.084 | 0.183 |
| parahippocampal | 2.699 | -0.264 | 5.481 | 0.073 | 0.128 |
| paracentral | -0.255 | -1.854 | 1.545 | 0.737 | 0.839 |
| pars opercularis | 0.625 | -0.666 | 2.100 | 0.357 | 0.480 |
| pars orbitalis | 0.082 | -1.661 | 1.850 | 0.922 | 0.983 |
| pars triangularis | 1.200 | -0.168 | 2.608 | 0.092 | 0.188 |
| pericalcarine | -0.330 | -1.783 | 1.034 | 0.654 | 0.762 |
| postcentral | 0.706 | -0.941 | 2.369 | 0.400 | 0.683 |
| posterior cingulate | -0.545 | -2.398 | 1.427 | 0.543 | 0.599 |
| precentral | 0.485 | -1.263 | 2.208 | 0.638 | 0.744 |
| precuneus | 0.096 | -1.289 | 1.573 | 0.899 | 0.899 |
| rostral anterior cingulate | -2.160 | -4.633 | 0.202 | 0.085 | 0.305 |
| rostral middle frontal | 0.261 | -0.853 | 1.362 | 0.672 | 0.672 |
| superior frontal | 0.382 | -0.949 | 1.808 | 0.619 | 0.619 |
| superior parietal | 0.361 | -1.222 | 1.988 | 0.631 | 0.883 |
| superior temporal | 0.602 | -1.143 | 2.495 | 0.553 | 0.645 |
| supramarginal | 0.658 | -0.820 | 2.271 | 0.366 | 0.366 |
| frontal pole | 1.061 | -1.605 | 3.667 | 0.416 | 0.564 |
| temporal pole | 2.561 | -0.758 | 6.296 | 0.133 | 0.233 |
| transverse temporal | 1.499 | -1.154 | 4.242 | 0.255 | 0.504 |
| insula | -0.105 | -1.798 | 1.490 | 0.889 | 0.931 |

**Supplemental Table 17. Effect modification of inferior temporal FTP on the relationship between LC MRI signal intensity and cortical thickness for the low beta-amyloid sample, corrected for age, sex, and years of education.** Analyses were performed using bootstrapped linear regressions with cortical thickness regions as the dependent variable, LC MRI signal intensity interacted with inferior temporal FTP as the independent variables, and age, sex and years of education as covariates (n = 112). Reported regression coefficients are unstandardized, and P-values have been corrected for multiple comparisons using FDR. Significant P-values have been indicated in bold. Significant P-values of regions in models with non-PVC data have been outlined by a blue box. In individuals with low beta-amyloid, lower LC MRI signal intensity is associated with lower entorhinal cortical thickness, particularly in individuals with elevated inferior temporal FTP. Abbreviations: CI, confidence interval; FDR, False Discovery Rate; Bankssts, banks of superior temporal sulcus.

| **Cortical thickness region** | ***B*** | **Lower 95% CI** | **Upper 95% CI** | ***P*-value** | ***P_FDR_*-value** |
| --- | --- | --- | --- | --- | --- |
| bankssts | 1.113 | -0.424 | 2.825 | 0.137 | 0.272 |
| caudal anterior cingulate | -0.176 | -2.488 | 2.079 | 0.903 | 0.932 |
| caudal middle frontal | 0.140 | -1.106 | 1.441 | 0.837 | 0.937 |
| cuneus | 0.202 | -0.959 | 1.400 | 0.762 | 0.889 |
| entorhinal | 5.910 | 3.009 | 8.572 | **0.000** | **0.000** |
| fusiform | 1.286 | 0.078 | 2.439 | **0.038** | 0.070 |
| inferior parietal | 1.101 | 0.037 | 2.283 | **0.041** | 0.071 |
| inferior temporal | 0.693 | -0.463 | 1.871 | 0.243 | 0.431 |
| isthmus cingulate | 2.005 | 0.262 | 3.767 | **0.023** | **0.040** |
| lateral occipital | 1.472 | 0.454 | 2.528 | **0.005** | **0.009** |
| lateral orbitofrontal | 0.850 | -0.275 | 2.059 | 0.138 | 0.242 |
| lingual | 0.675 | -0.366 | 1.711 | 0.205 | 0.359 |
| medial orbitofrontal | 1.244 | -0.185 | 2.566 | 0.084 | 0.205 |
| middle temporal | 1.145 | -0.011 | 2.270 | 0.055 ^ | 0.127 |
| parahippocampal | 1.617 | -0.947 | 4.136 | 0.214 | 0.327 |
| paracentral | -0.588 | -1.959 | 0.868 | 0.394 | 0.528 |
| pars opercularis | 0.137 | -0.994 | 1.269 | 0.793 | 0.941 |
| pars orbitalis | 0.677 | -0.773 | 2.120 | 0.366 | 0.512 |
| pars triangularis | 0.927 | -0.277 | 2.040 | 0.129 | 0.252 |
| pericalcarine | 0.304 | -0.773 | 1.358 | 0.574 | 0.574 |
| postcentral | 0.896 | -0.499 | 2.336 | 0.202 | 0.353 |
| posterior cingulate | 0.127 | -1.262 | 1.748 | 0.915 | 0.929 |
| precentral | 0.112 | -1.300 | 1.546 | 0.898 | 0.916 |
| precuneus | -0.211 | -1.339 | 1.009 | 0.719 | 0.997 |
| rostral anterior cingulate | -0.746 | -2.745 | 1.193 | 0.502 | 0.846 |
| rostral middle frontal | 0.297 | -0.719 | 1.383 | 0.534 | 0.606 |
| superior frontal | -0.050 | -1.139 | 1.242 | 0.902 | 0.905 |
| superior parietal | 0.369 | -0.966 | 1.675 | 0.577 | 0.680 |
| superior temporal | 0.614 | -0.706 | 2.205 | 0.410 | 0.573 |
| supramarginal | 0.753 | -0.449 | 1.994 | 0.243 | 0.283 |
| frontal pole | 1.682 | -0.802 | 3.789 | 0.133 | 0.234 |
| temporal pole | 3.067 | 0.512 | 5.902 | **0.018** | **0.032** |
| transverse temporal | 0.914 | -1.303 | 3.198 | 0.417 | 0.641 |
| insula | 0.188 | -1.195 | 1.637 | 0.838 | 0.895 |

**Supplemental Table 18. Effect modification of entorhinal FTP on the relationship between LC MRI signal intensity and cortical thickness for the CDR = 0 sample with low beta-amyloid, corrected for age, sex, and years of education.** Analyses were performed using bootstrapped linear regressions with cortical thickness regions as the dependent variable, LC MRI signal intensity interacted with EC FTP as the independent variables, and age, sex and years of education as covariates (n = 112). Reported regression coefficients are unstandardized, and P-values have been corrected for multiple comparisons using FDR. Significant P-values have been indicated in bold, and P-values at trend level are indicated with ^. Significant P-values of regions in models with non-PVC data have been outlined by a blue box. In individuals with CDR = 0 and low beta-amyloid, lower LC MRI signal intensity is associated with lower entorhinal, isthmus cingulate, lateral occipital and temporal polar cortical thickness, particularly in individuals with elevated EC FTP. Abbreviations: CI, confidence interval; FDR, False Discovery Rate; Bankssts, banks of superior temporal sulcus.
